# Ca^2+^-pumping by PMCA-neuroplastin complexes operates in the kiloHertz-range

**DOI:** 10.1038/s41467-025-62735-5

**Published:** 2025-08-20

**Authors:** Cristina E. Constantin, Barbara Schmidt, Yvonne Schwarz, Harumi Harada, Astrid Kollewe, Catrin S. Müller, Sebastian Henrich, Botond Gaal, Akos Kulik, Dieter Bruns, Uwe Schulte, Heiko Rieger, Bernd Fakler

**Affiliations:** 1 Institute of Physiology, Faculty of Medicine, University of Freiburg, Freiburg, Germany; 2 Center for Biophysics & Department of Physics, Saarland University, Saarbrücken, Germany; 3 Institute of Physiology, Center for Integrative Physiology and Molecular Medicine, Saarland, Homburg, Germany; 4 Department of Anatomy, Histology and Embryology, Faculty of Medicine, University of Debrecen, Debrecen, Hungary; 5 Logopharm GmbH, March-Buchheim, Germany; 6 Signalling Research Centres BIOSS and CIBSS, Freiburg, Germany; 7 Center for Basics in NeuroModulation, Freiburg, Germany

**Keywords:** Calcium signalling, Transporters in the nervous system

## Abstract

Ca^2+^-ATPases in the plasma membrane extrude Ca^2+^ ions from the cytosol to the extracellular space thereby terminating Ca^2+^-signals and controlling Ca^2+^-homeostasis in any type of cell. Recently, these Ca^2+^-pumps have been identified as protein complexes of the transporting subunits PMCAs1-4 and the single-span membrane proteins Neuroplastin (NPTN) or Basigin that are obligatory for efficient trafficking of the pump complexes to the surface membrane. Quantitative investigation of the pumping velocity controlling the time course of Ca^2+^-signals, however, has remained unresolved. Here we show, using Ca^2+^-activated K^+^ channels as fast native reporters of intracellular Ca^2+^ concentration(s) together with membrane-tethered fluorescent Ca^2+^-indicators, that under cellular conditions PMCA2-NPTN complexes can clear Ca^2+^ in the low millisecond-range. Computational modeling exploiting EM-derived densities of Ca^2+^-source(s) and Ca^2+^-transporters in freeze-fracture replicas translated these fast kinetics into transport rates for individual PMCA2-NPTN pumps of more than 5000 cycles/s. Direct comparison with the Na^+^/Ca^2+^-exchanger NCX2, an alternate-access transporter with established cycling rates in the kHz range, indicated similar efficiencies in Ca^2+^-transport. Our results establish PMCA2-NPTN complexes, the most abundant Ca^2+^-clearing tool in the mammalian brain, as transporters with unanticipated high cycling rates and demonstrate that under cellular conditions ATPases may operate in the kHz-range.

## Abstract (web summary)

Ca2 + -ATPases in the plasma membrane extrude Ca2+ ions from the cytosol to the extracellular space and thus terminate Ca2 + -signals and control Ca2 + -homeostasis. Here, Constantin et al. show that these pumps operate at rates of more than 5000 cycles/s.

## Introduction

The calcium (Ca^2+^) transporting ATPases of the plasma membrane (PMCAs) are P-type ATPases or pumps that are expressed in virtually any type of cell where they extrude Ca^2+^ from the cytosol to the extracellular space using ATP as an energy source^1–3^. This extrusion is crucial for the termination of Ca^2+^-signals, transient increases in intracellular Ca^2+^ concentration ([Ca^2+^]_i_) that are used to precisely control a variety of fundamental cellular processes, including vesicle release, enzymatic activity, excitability, contraction, and motility^4–6^.

Molecularly, native PMCAs have recently been identified as heteromeric complexes of the ATPase subunits PMCAs1–4 and the single-span membrane proteins Neuroplastin (NPTN) or Basigin (BASI) that co-assemble with the PMCA subunits in the ER and are required for stability and proper trafficking of the Ca^2+^-pump complexes^7–10^.

In neurons, genetic or pharmacological ablation of the PMCA-mediated transport demonstrated that Ca^2+^ extrusion occurs within tens of milliseconds or faster suggesting that individual Ca^2+^-pumps operate with rapid transport rates in the cellular context^10–12^. As yet, however, determination of these transport rates has been hampered by technical limitations precluding accurate assessment of both transport activity and number of active Ca^2+^-pumps involved.

Here, we use time-resolved monitoring of Ca^2+^-transport and changes in [Ca^2+^]_i_ in intact cells together with EM-based protein counting in the plasma membrane to show that individual PMCA2-NPTN complexes operate at cycle-rates in the kHz-range and thus enable Ca^2+^-signaling with millisecond precision.

## Results

### Fast and effective Ca^2+^-clearing by PMCA-NPTN pumps

**Fig. 1:**
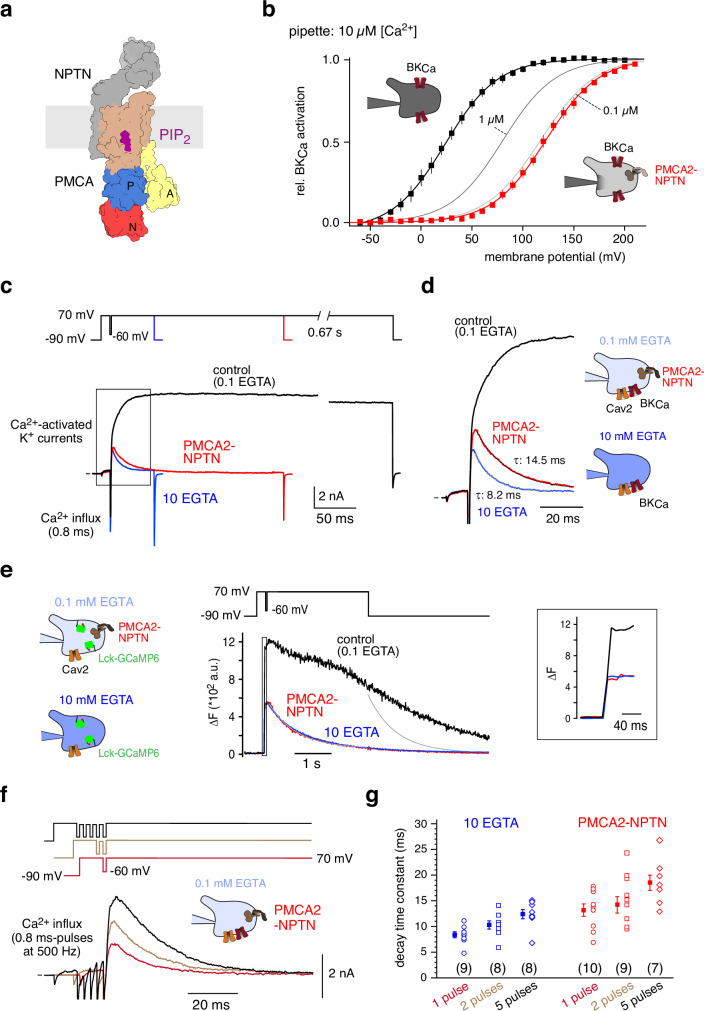
Ca^2+^-clearance by PMCA2-NPTN in the millisecond-range. **a** Surface-illustration (space-filling mode) of the PMCA2-NPTN complex with bound PIP_2_ as determined in cryo-EM analyses^16^. **b** Steady-state activation curves of BK_Ca_ channels recorded in whole-cell mode with 10 µM free Ca^2+^ in the patch-pipette (insets) from CHO(ΔPMCA2)-cells in the absence (squares and line in black, 8 cells) and presence of heterologously expressed PMCA2-NPTN complexes (squares and line in red, 11 cells). Activation curves recorded with 1 µM and 0.1 µM free Ca^2+^ in the patch-pipette were added for calibration. Lines are result of a Boltzmann function fitted to the data (mean ± SEM). Note the large pump-mediated shift of the activation curve indicating a reduction of [Ca^2+^]_i_ from pipette-delivered 10 µM (dark grey) to values below 0.1 µM (light grey). **c** Representative BK_Ca_-mediated outward K^+^ currents recorded with the indicated voltage-protocol in cultured CHO cells in response to a 0.8 ms Ca^2+^ influx through voltage-gated Cav2.2 channels in the presence of either 10 mM EGTA (trace in blue) or 0.1 mM EGTA in the absence (control, black trace) or presence of PMCA2-NPTN complexes (trace in red). Note the rapid current decay with 10 mM EGTA and with PMCA2-NPTN at 0.1 mM EGTA as a cytoplasmic buffer. **d** Current traces (in the framed box in (**c**)) at an expanded time scale, the decay phase approximated with a mono-exponential function with indicated time constant (τ). Currents were scaled to the maximum prior to the Ca^2+^ influx pulse. **e** Representative fluorescence traces recorded in 10-12 experiments with identical conditions as in (**c**), but with the membrane-tethered fluorescent Ca^2+^-indicator Lck-GCaMP6s used for monitoring changes in [Ca^2+^]_i_ (left insets). Gray line is the result of a mono-exponential fitted to the decay phase of the traces with PMCA2-NPTN and 10 EGTA. Right inset: Fluorescent signals (at the framed box) at an expanded time scale. **f** Representative BK_Ca_-currents recorded in response to 1, 2 and 5 Ca^2+^ influx pulses applied at 500 Hz (voltage protocol in inset) to CHO cells as in (**a**). **g** Plot summarizing the time constants of the current decay determined in experiments as in (**f**); squares represent mean ± SEM of the indicated number of cells.

The Ca^2+^-pump activity of PMCAs critically depends on intact cellular boundaries, in particular sufficient supply of ATP and physiological levels of phospholipids (including PIP_2_ (phosphatidylinositol-4,5-bisphosphate)) in the plasma membrane^13–15^. In fact, our structural analysis of PMCA2-NPTN complexes by cryo-EM identified a PIP_2_ molecule that is inserted into a pocket close to the Ca^2+^-passageway and that is obligatory for the transport function of the pump complex (Fig. 1a^16^). For investigating the Ca^2+^-transport in PMCA-NPTN complexes we, therefore, turned to heterologous expression in CHO cells, an epithelial line established for analysis of membrane proteins with phospholipid-dependence^17–21^. To reliably determine transport function in intact cells, we chose to monitor intracellular Ca^2+^ ([Ca^2+^]_i_, rather than ATP-consumption or pH-changes resulting from the Ca^2+^−2H^+^ countertransport) using co-expressed BK-type Ca^2+^-activated K^+^ channels (BK_Ca_, homomeric BKα, see Methods) as native sensors. These channels sense apparent [Ca^2+^]_i_ in the physiological range (0.1–10 µM^22^) at the plasma membrane and convert changes in [Ca^2+^]_i_ into changes in channel gating (and K^+^ currents) with ms-resolution (Supplementary Fig. 1^22–24^). Importantly, BK_Ca_ channels do not interfere with [Ca^2+^]_i_ as they do not effectively act as buffers (due to their low number, in contrast to soluble (fluorescent)-indicators that depend on stoichiometric Ca^2+^ binding).

Three types of experiments were used to analyze PMCA2-NPTN-mediated Ca^2+^-transport in distinct cellular settings, with the first set investigating extrusion of Ca^2+^ ions against a high concentration of 10 µM free Ca^2+^ constantly infused into the cell by the de-facto unlimited reservoir of a whole-cell patch pipette (Fig. 1b, left inset). The BK_Ca_ activation curves recorded under these steady-state conditions in the absence (control) and presence of the pump complexes were largely different: PMCA2-NPTN fueled by 2.5 mM ATP (through the patch pipette) induced an extensive right-shift of the activation curve compared to control, placing it slightly below the calibration curve determined with 0.1 µM free Ca^2+^ and thus indicating a pump-mediated decrease in [Ca^2+^]_i_ at the plasma membrane of more than 2 orders of magnitude (Fig. 1b, right inset).

In a second set of experiments, the pumps were challenged with a 0.8 ms-‘[Ca^2+^]_i_-step’ generated by a voltage-commanded Ca^2+^ influx through stably expressed N-type voltage-gated Ca^2+^ (Cav2.2) channels (see Methods). In CHO cells perfused with 0.1 mM EGTA, the equivalent of cytoplasmic Ca^2+^-buffering in most CNS neurons^25^, such an action potential-like Ca^2+^ influx resulted in a lasting increase in [Ca^2+^]_i_ reflected by activation of persistent (slowly decaying) BK_Ca_-mediated outward K^+^ currents (Fig. 1c, control trace). In contrast, when EGTA (in the perfusing pipette solution) was increased to 10 mM, BK_Ca_-currents activated by the same brief Ca^2+^ influx displayed a rapid decay (Fig. 1c, d, traces in blue) with time constants (τ _decay_) of ∼8 ms (mean ± SEM: 8.4 ± 0.6 ms, n = 9, Fig. 1g). This τ _decay_ perfectly matched the time constant of channel deactivation (at 50 mV, Supplementary Fig. 1a) indicating complete absorption of the incoming Ca^2+^ ions by EGTA-buffering within less than a millisecond^23^. Surprisingly, Ca^2+^-triggered BK_Ca_-currents with very similar decay kinetics could also be recorded at low intracellular EGTA of 0.1 mM upon co-expression of PMCA2-NPTN complexes (Fig. 1c, d, traces in red). The respective value for τ _decay_ was ∼13 ms (mean ± SEM: 13.2 ± 1.2 ms, n = 10, Fig. 1g) indicative of pump-mediated removal of Ca^2+^ at a speed resembling that by buffering with 10 mM EGTA. These conclusions were further corroborated by measuring the [Ca^2+^]_i_ transients in the same step experiments with the membrane-tethered Lck-GCaMP6 (Supplementary Fig. 2, Methods^26,27^;) replacing BK_Ca_ channels as Ca^2+^-indicators: The Ca^2+^-triggered fluorescence signal, after an initial increase (Fig. 1e, right panel), displayed complete decay with both 10 mM EGTA (in the cytoplasm) or PMCA2-NPTN co-expression (at low intracellular EGTA of 0.1 mM), but persisted and declined slowly under control conditions with 0.1 mM EGTA in the cytoplasm (Fig. 1e). The respective τ_decay_ of ∼740 ms determined with 10 mM EGTA and PMCA2-NPTN co-expression closely matched the dissociation-time measured for Ca^2+^ ions from GCaMP6s in stopped-flow fluorimetry with one ms-mixing^26^, supporting the fast Ca^2+^-clearing determined with BK_Ca_ channels before (Fig. 1c, d).

In a third set of experiments, Ca^2+^ extrusion by either PMCA2-NPTN (at 0.1 mM EGTA) or 10 mM EGTA was further probed in experiments where two or five 0.8 ms-pulses of Ca^2+^ influx were elicited at a frequency of 500 Hz. These additional Ca^2+^ pulses interrupted by 2 ms non-influx intervals (Fig. 1f, upper inset) led to an increased [Ca^2+^]_i_ as reflected by the concomitant increase of the BK_Ca_-current maxima. The subsequent Ca^2+^-clearing, however, appeared only slightly prolonged in either case (Fig. 1f, Supplementary Fig. 3). The latter is indicated by the respective increase in τ _decay_ (from 8.4 to 12.4 ms (10 EGTA), and from 13.2 to 18.5 ms (PMCA2-NPTN); Fig. 1f, g; Supplementary Fig. 3b). Interestingly, [Ca^2+^]_i_ did not prominently increase with the number of Ca^2+^ pulses, pointing to sufficient supply of Ca^2+^-free EGTA and to fast extrusion of Ca^2+^ by the pump complexes during the 2-ms non-influx intervals, respectively.

Together, the results of all three sets of experiments unequivocally indicated that PMCA2-NPTN complexes can terminate increased [Ca^2+^]_i_ in the ms-range as already noticed in some neurons^10–12^, and raised the fundamental question whether this fast Ca^2+^ extrusion may be due to fast transport of individual pump complexes or rather result from a high-density expression in the plasma membrane combined with slow pumping activity.

### Membrane densities and amounts of PMCA pumps in cells and tissues

**Fig. 2:**
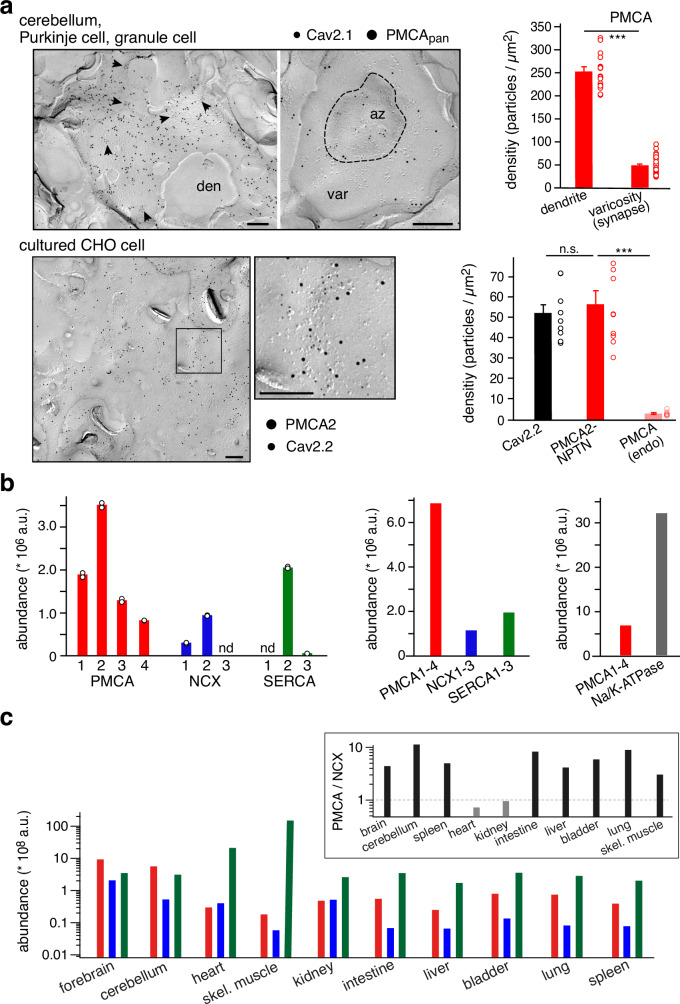
Expression and surface density of PMCA-NPTN complexes in mouse brain and CHO cell testing system. **a** Upper panel: Left, Electron micrographs showing distribution of immunogold particles for Cav2.1 channels (6 nm-gold particles) and PMCA1-4 proteins (12 nm-gold particles) on dendritic shafts of Purkinje cells (den) and in presynaptic varicosities (var) of granule cells in the cerebellum. Dashed line demarks the active zone (az); arrowheads depict clusters of Cav2.1 channels. Scale bars represent 200 nm. Right: Graph denoting the density of PMCAs1-4 in Purkinje cell dendrites and granule cell varicosities (bars are mean ± SEM of the 19 dendrites and 37 varicosities shown). Lower panel: Left, Distribution of Cav2.2 channels (6 nm-gold particles) and PMCA2 protein (12 nm-gold particles) on the plasma membrane of CHO cells that stably express Cav2.2 (α1, β1, α2δ1) and that were transiently transfected with PMCA2 and NPTN. Inset: The framed box on the left at expanded scale. Scale bars represent 200 nm. Right: Graph summarizing surface density for the indicated proteins; values are mean ± SEM of 10 (Cav2.2, PMCA2) and 12 (endo) cells, respectively. Statistical testing between groups of data are indicated by horizontal bars, three stars denote p-values < 0.001, Mann-Whitney U-test (two-sided), n.s. is not significant (exact p-values were 0.0000001 (dendrite vs varicosity), 0.00076 (PMCA-NPTN vs PMCA(endo)). **b** Molecular abundance of the Ca^2+^-transporting proteins PMCAs1-4, NCXs1-3 and SERCAs1-3 determined by quantitative mass spectrometry in membrane fractions from pooled whole mouse brains. Bars denote mean values of the duplicate experiments shown. Amounts determined for alpha subunits 1-3 of the Na/K-ATPase were added for comparison. Note that PMCAs are the most abundant Ca^2+^-transporting system in the brain. n.d. no detected. **c** Molecular abundance of all transporting subunits of the Ca^2+^-pumps in plasma membrane and membrane of the sarco-/endoplasmic reticulum, as well as of the Na^+^/Ca^2+^-exchanger in the distinct tissues/organs indicated (skel. is skeletal). Abundance scaling is logarithmic; all protein amounts were determined in equivalent amounts of membrane(s). Note that PMCA pumps outnumber the exchangers in all tissues except heart and kidney (inset), and that the brain is the only organ where the amounts of PMCAs exceed that of the SERCAs.

In pursuit of this question, we next used immunogold electron-microscopy (immuno-EM) on SDS-digested freeze-fracture replicas (SDS-FRL) of brain neurons and CHO cells, as well as quantitative mass spectrometry (MS) on membrane fractions from various tissues. Labelling with a PMCA-specific antibody (PMCA_pan_, see Methods) at saturating concentration showed that PMCAs1-4 are distinctly distributed over the surface membrane of different brain neurons. Thus, on dendrites of cerebellar Purkinje cells the density of PMCAs amounted to ∼250 µm^2^ (equivalent to more than 10% of all intra-membraneous particles), while roughly 50 PMCAs/µm^2^ were found in the active zone of parallel fiber varicosities (or pre-synaptic terminals) of the granule cell-to-Purkinje-cell synapses (Fig. 2a, upper panel). Application of the PMCA_pan_ antibody to freeze-fracture replicas from CHO cells expressing PMCA2-NPTN complexes indicated a density of ∼55 pump complexes per µm^2^ exceeding the endogenous levels of PMCAs in these epithelial cells by more than ten-fold (Fig. 2a, lower panel). In the same CHO cells the density of stably expressed Cav2.2 channels stained with a target-specific Cav2.2 antibody (Methods) exhibited a mean value of ∼52/µm^2^ (Fig. 2a, lower panel) thus demonstrating about equal amounts for both, Ca^2+^-source and Ca^2+^-transporter, in our testing system (Fig. 1). Noteworthy, the majority of the Cav2.2 immuno-particles appeared in clusters (of up to 10 proteins), while the PMCA-particles were more homogenously distributed; preferred co-clustering between both Cav2.2 and PMCA2-NPTN was not observed.

In addition, we assessed the amounts of Ca^2+^-transport systems in brain and other tissues of adult mice by quantitative MS on integral membrane fractions (^28,29^, Methods). These analyses unraveled PMCAs as the most abundant Ca^2+^-transporters in the brain exceeding both the Na^+^/Ca^2+^-exchangers (NCX) and the Ca^2+^-pumps of the endo/sarcoplasmic reticulum (SERCA) by several-fold and indicated that PMCA2 exhibits highest expression among the four PMCA proteins (Fig. 2b, Supplementary Fig. 4). Conversely, PMCA-pumps are largely outnumbered (more than five-fold) in brain membranes by the Na/K-ATPase, another P-type ATPase in the plasma membrane (Fig. 2b, right panel). Interestingly, the observed excess of PMCAs over both NCX and SERCA is a unique feature of the brain (forebrain and cerebellum), while in all other tissues analyzed, SERCA is the most abundant Ca^2+^-transport system. In these tissues PMCA dominated over NCX in the surface membrane, except for heart and kidney where the PMCA-to-NCX ratio was slightly less than one (values of 0.75 and 0.95, respectively; Fig. 2c, inset).

### Computational modelling of transport cycles in PMCA pumps

**Fig. 3:**
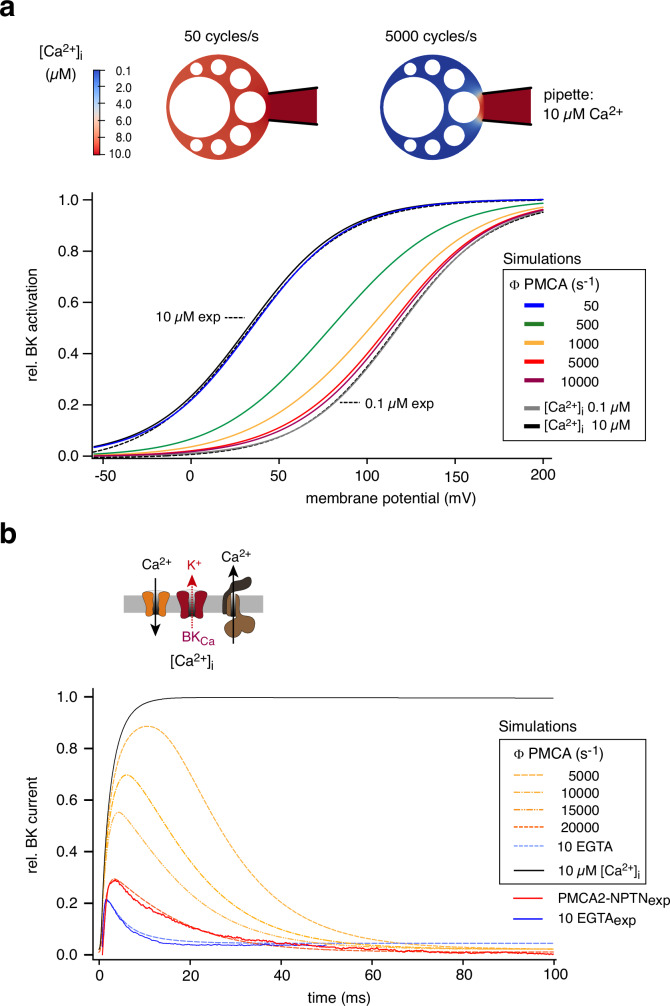
Determination of transport rates in PMCA2-NPTN complexes. **a** Computed activation curves of BK_Ca_ channels (using the stationary model, Supplementary Table 2) determined with 10 µM free Ca^2+^ delivered through the patch-pipette in the absence (line in black) and presence of PMCA2-NPTN operating at the indicated rates (colored lines). The computed activation for 0.1 µM [Ca^2+^]_i_ (line in gay), and the experimentally determined activation curves for [Ca^2+^]_i_ of 10 µM and 0.1 µM (dashed lines) are indicated. Inset: [Ca^2+^]_i_ profiles obtained with constant Ca^2+^ influx (10 µM) from the pipette and Ca^2+^ outflux mediated by PMCA complexes transporting at rates of 50 and 5000 cycles/s; color-coding of [Ca^2+^]_i_ as indicated. Note close overlay between computed and experimental data for pipette-set [Ca^2+^]_i_ of 10 µM and 0.1 µM (calibration), and of computed curves for [Ca^2+^]_i_ of 10 µM with PMCA2-NPTN operating at transport rates of 5−10.000 cycles/s with experimental curves recorded with pipette-set [Ca^2+^]_i_ of 0.1 µM. **b** Computed responses of BK_Ca_ channels (using the non-stationary model, Supplementary Table 3) to pulsed increases in [Ca^2+^]_i_ either with PMCA2-NPTN operating at the indicated rates (dashed lines in yellow/orange) or with 10 mM EGTA as a cytoplasmic buffer (dashed lines in blue). BK_Ca_-currents experimentally determined with either 10 mM EGTA or PMCA2-NPTN are indicated as lines in red and blue, respectively. Black trace is the model response to a homogenous increase of [Ca^2+^]_i_ from 0.1 to 10 µM in the absence of PMCA pumps.

With the precise data acquired on [Ca^2+^]_i_ dynamics and BK_Ca_-gating (Fig. 1), as well as on densities of PMCA2-NPTN complexes and Cav2.2 channels in the plasma membrane of CHO cells (Fig. 2), we next turned to computational modeling for determining the rates of Ca^2+^ -transport in individual pump complexes. For realistic representation of the experimental conditions, the following procedures were applied: (1) BK_Ca_-gating was described by the response of a well-established 10-state model^30^ whose transition rates were calibrated by machine-learning procedures using experimental data with defined variations in [Ca^2+^]_i_ and transmembrane voltage as an input (Methods, Supplementary Tables 1-3, Supplementary Fig. 5a, b^23^,); (2) spatio-temporal profiles for [Ca^2+^]_i_ were determined in a three-dimensional diffusion model (of a spherical cell) without and with Ca^2+^ efflux mediated by transport of the (homogenously distributed) PMCA2-NPTN pumps at defined density (50 µm^2^), but variable cycling rates (Fig. 3a, inset); (3) Ca^2+^ influx was either stationary through a single reservoir (pipette) or transient (0.8 ms) through a density-defined number of Cav channels (50 µm^2^) providing an experimentally determined mean number of Ca^2+^ ions (equations and parameters used are detailed in Methods and Supplementary Tables 1-3).

In a first step, we recapitulated the pump-mediated shift in activation curves (Fig. 1b) by computing the response of the calibrated BK_Ca_ model to [Ca^2+^]_i_ profiles obtained with constant Ca^2+^ influx (10 µM) from the pipette and increasing cycle rates for the PMCA complexes. As illustrated in Fig. 3a, the resulting BK_Ca_ response(s) perfectly matched the experimentally determined calibration curves ([Ca^2+^]_i_ of 10 and 0.1 µM in the absence of pumps, dashed lines in Fig. 3a), and showed that increasing cycle rates of the Ca^2+^-pumps shifted BK_Ca_ activation towards positive membrane potentials (Fig. 3). Interestingly, the experimentally observed shift generated by PMCA2-NPTN required the pumps to operate at rates of ≥5.000/s (Fig. 3a). Currently assumed pump rates of 50 s^31^ failed to produce a visible shift of the activation curve.

In a second step, we modelled the BK_Ca_ current-transients recorded upon the pulsed increases in [Ca^2+^]_i_ in the presence of either high buffer concentrations or PMCA complexes (Fig. 3b, traces in blue and red, respectively). In these calculations, [Ca^2+^]_i_ at the end of the 0.8 ms-influx pulse was 10 µM, a minimal value to account for the observed rapid onset of the Ca^2+^-activated BK_Ca_ currents^23^, while the resting [Ca^2+^]_i_ was set to 0.1 µM. The response determined with 10 mM EGTA as a cytoplasmic Ca^2+^-buffer (with known kinetics^32^) was used for validation of the model’s performance. As illustrated in Fig. 3b, the experimentally determined current traces were closely approximated by the responses of the BK_Ca_ model when [Ca^2+^]_i_ was removed either by EGTA (dashed line in blue) or by PMCA2-NPTN operating at a rate of ∼20.000 s (dashed line in orange). Lower transport rates failed to match the experimental traces with respect to both peak-current (time to amplitude maximum) and decay kinetics of the current traces (Fig. 3b, dashed traces in yellow; Supplementary Fig. 5c).

Together, our modelling data strongly suggest that native Ca^2+^-pumps transport their substrate with rates in the kHz-range, very similar to what was previously reported for native and heterologously expressed NCX-type Ca^2+^-transport proteins^33^.

### Comparison of Ca^2+^-transport by PMCA-NPTN and NCX

**Fig. 4:**
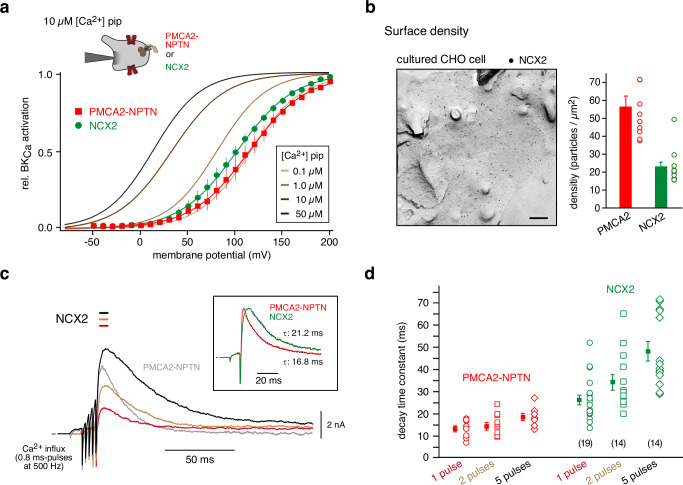
Comparison of Ca^2+^-clearing by NCX2 and PMCA2-NPTN complexes expressed in CHO cells. **a** Activation curve of BK_Ca_ channels recorded in whole-cell mode with 10 µM free Ca^2+^ in the patch pipette (inset) is distinctly shifted to the right by co-expression of either PMC2-NPTN (red symbols, 12 cells) or NCX2 (green symbols, 14 cells). Activation curves determined in experiments with the indicated [Ca^2+^]_i_ in the pipette are given as calibration for nominal intracellular Ca^2+^ concentrations (data from^10^). Lines are result of a Boltzmann function fitted to the data (mean ± SEM). Note the large shift of the activation curve indicating a reduction of [Ca^2+^]_i_ from pipette-delivered 10 µM (dark grey) to values around 0.1 µM (light grey) by both Ca^2+^-transporters. **b** Electron micrograph showing distribution of immunogold particles for NCX2 transiently expressed in CHO cells and bar graph (mean ± SEM of the 13 cells shown) denoting surface density of the NCX2 protein. Data for PMCA2 from Fig. 2a were added for comparison. **c** Representative BK_Ca_-currents recorded with 0.1 mM EGTA as a cytoplasmic buffer in response to 1, 2, and 5 Ca^2+^ influx pulses as in Fig. 1f in CHO cells expressing Cav2.2 and NCX2. The current-response to 5 pulses obtained with PMCA2-NPTN from Fig. 1f (trace in grey) was added for comparison. Inset: BK_Ca_-currents recorded upon a single 0.8 ms Ca^2+^ influx pulse with NCX2 or PMCA2-NPTN. Note slightly faster decay of the BK_Ca_-currents in the presence of PMCA2-NPTN complexes. **d** Plot summarizing the time constants of the current decay determined in experiments as in (**c**); squares represent mean ± SEM of the indicated number of cells; data for PMCA2-NPTN were added for comparison.

We, therefore, thought to probe this prediction by direct comparison between both types of transporters in an identical experimental setting. We recapitulated the experiments in Fig. 1b, c with NCX2, the most abundant NCX isoform of the rodent brain (Fig. 2b), replacing PMCA2-NPTN complexes. With NCX2 present in the plasma membrane, the steady-state activation curve of BK_Ca_ channels measured with a [Ca^2+^]_pip_ of 10 µM was shifted to the right indicating that NCX2 decreased [Ca^2+^]_i_ underneath the plasma membrane into the sub-micromolar range similar to the activity of PMCA2-NPTN, albeit somewhat less effective (Fig. 4a). Subsequent probing of the speed of Ca^2+^ extrusion by NCX2 with short Ca^2+^ influx pulses as in Fig. 1c showed that the exchanger promoted a rapid decay of the BK_Ca_ -currents with values for τ _decay_ of ∼26 ms (mean ± SEM: 26.2 ± 2.3 ms, *n* = 19). This two-fold slowing of the Ca^2+^-clearing compared to that by PMCA2-NPTN is in line with the about two-fold lower density of the NCX2 protein found by immuno-EM in freeze-fractures of the plasma membrane of the respective CHO cells (Fig. 4b). And, as a consequence of this slower Ca^2+^-clearing, high-frequency application of several voltage-commanded Ca^2+^ influx pulses led to a more prominent increase in the amplitude and the time-to-peak interval of the BK_Ca_-currents, as well as in the time constants of their decay (τ _decay_ increased from 26.1 to 48.2 ms) compared to PMCA2-NPTN-mediated Ca^2+^ removal in our test system (Fig. 4c, d). These results provided independent evidence that under cellular conditions PMCA2-NPTN complexes are able to transport Ca^2+^ with turnover rates at least in the range of 5000/s, a value previously determined for NCX in giant cell-attached patches from cardiomyocytes^33^.

## Discussion

Our work demonstrates that under cellular conditions PMCA2-NPTN complexes are able to remove increased intracellular Ca^2+^ with both high efficiency (against large high-concentration reservoirs) and high speed (within ms-windows). These transport characteristics are based on the pumps’ ability to operate in the kHz-range ( > 5000 cycles/s, Fig. 3) and thus at cycling rates similar to those previously reported for NCX2, an alternate access-type transporter for Ca^2+^. Direct comparison of both transporter types in identical experiments emphasized their similar Ca^2+^-transport capabilities.

For detailed investigation of the transport properties of PMCA pumps we set up experimental conditions that guaranteed maximal activity and its precise determination (in individual cells): (i) co-expression of NPTN promoted reliable and robust expression of PMCA pumps in the plasma membrane^9,10^, (ii) saturating physiological levels for both the phospholipid PIP_2_ and ATP were maintained throughout the experiments, (iii) [Ca]_i_ and its pump-mediated changes were monitored with BK_Ca_ channels, a native Ca^2+^-sensor operating in the physiologically-relevant [Ca^2+^]_i_-range with ms-precision and, importantly, without interfering with [Ca^2+^]_i_ (in contrast to soluble fluorescence-based reporter systems acting as additional Ca^2+^-buffers), and (iv) the number of PMCA pumps was determined by immuno-EM in freeze-fracture configuration that enables immediate and selective counting of specifically labelled target proteins in the membrane^34^. For labelling with an extended dynamic range as displayed by the PMCA_pan_ antibody (about three orders of magnitude, Fig. 2a), the protein counting obtained by this technique is highly accurate and reliable (as shown for ion channels^28,35,36^), in contrast to indirect approaches providing rather rough estimates on protein densities/numbers in membranes^31,37,38^.

The cycling rates in the kHz-range that were finally determined in computational modelling with the acquired experimental data as input are profoundly different from values of about 50–100/s that were estimated from transport-experiments with erythrocyte ghosts, or lipid vesicles containing endogenous or purified/reconstituted PMCA protein^31^. The reasons for these differences are most likely buried in the experimental settings and approaches used including detergent-mediated dissociation of PMCA and NPTN/BASI subunits^7,10^, erroneous data on transporter densities, and, most importantly, insufficient supply of PIP_2_ in the vesicle or ghost preparations. As indicated by structure-function analyses, PIP_2_-binding is obligatory for the transport activity of PMCA pumps^16^. Conversely, transport at high cycling rates could be well observed in intact erythrocytes: While different in settings and dimensions, the measured transport-associated ATP-consumption is compatible with the kHz-rates derived in our experiments (60 mmoles/hl^39^ versus ∼120 mmoles/hl calculated with a turnover rate of 5000/s and an estimated PMCA-density of 6/µm^2^).

The finding of PMCA2-NPTN-mediated Ca^2+^ extrusion occurring in the ms-range has fundamental implications for both the amplitude and the time course of Ca^2+^-signaling (or increased [Ca^2+^]_i_) in virtually any type of cell. In CNS neurons, where fast pump-mediated Ca^2+^-clearing is well known^11,12,40^ and where PMCA-NPTN is the most abundant Ca^2+^-clearing device (Fig. 2b, c), rapid termination of increased [Ca^2+^]_i_ and prevention of Ca^2+^ accumulation is prerequisite for reliable and timely accurate neurotransmission in both dendrites and synapses^10,11,40–42^. In synapses, PMCA-NPTN complexes are expected to act in concert with cytoplasmic Ca^2+^-buffers to effectively promote synchronous over asynchronous vesicle release even at high frequency transmission (100 to more than 1000 Hz)^25,43,44^. For this purpose, the increases in [Ca^2+^]_i_ fueled by action potential-triggered activation of Cav channels must be rapidly cleared. Explicitly, the about 200 Ca^2+^ ions delivered by any Cav2 channel per millisecond must be effectively extruded (to prevent buffer saturation), with PMCA pumps contributing at a rate of 10 Ca^2+^ ions/ms based on a cycling rate of 10 kHz (Figs. 1f, 3).

To what extent the fast Ca^2+^ extrusion by PMCA-NPTN participates in other signaling processes in the CNS, as well as in other Ca^2+^-driven second-messenger reactions should be (re)-investigated in detail, best by acute removal of all Ca^2+^-ATPases in the plasma membrane as can be achieved by knock-out/down of their obligatory auxiliary subunits NPTN and BASI^10^. Moreover, it appears worthwhile to investigate the relation between NCX and PMCA-NPTN, as both transport systems are able to drive fast reduction of activity-induced increases in [Ca^2+^]_i_ to resting levels of about 100 nM (Figs. 1, 4).

## Methods

### Ethical Information

Ethical approval was provided by Regierungspräsidium Freiburg (Germany).

### Cell culture and transfection

CHO cells (in wildtype form) and CHO cells stably expressing Cav2.2 channels (Cav2.2α1; Cavβ1, α2δ1 subunits) were transiently transfected with cDNAs having the following GenBank (https://www.ncbi.nlm.nih.gov/genbank) accession numbers: Q08460 (BK_Ca_), NM_001036684.3 (PMCA2), NM_009145.2 (Neuroplastin), NM_078619.1 (NCX2). The fusion proteins of PMCA2 or NCX2 with green fluorescent protein as well as Neuroplastin with red fluorescence protein were prepared and verified by sequencing. Cells were incubated at 37 °C and 5% CO_2_ and measured 2 to 4 days after transfection. Lck-GCamp6s (addgene #52924) was subcloned into the eukaryotic expression vector pN1-CMV, verified by sequencing and used for transfection using the standard lipofectamine 2000 protocol; recordings were done 30–36 h after transfection.

### Electrophysiology

Whole-cell patch clamp recordings were performed at room temperature using a HEKA EPC 10 amplifier. Currents were low-pass-filtered at 3–10 kHz and sampled at 20 kHz. Leak currents were subtracted with a P/4 protocol at a holding potential of −90 mV. Serial resistance was 50–70% compensated using the internal compensation circuitry. The standard extracellular solution contained (in mM): KCl 5.8; NaCl 144; MgCl_2_ 0.9; CaCl_2_ 1.3; NaH_2_PO_4_ 0.7; D-Glucose 5.6, and HEPES 10 (pH 7.4). Recording pipettes pulled from quartz glass had resistances of 2–3.5 MΩ when filled with internal solution.

For recordings of BK_Ca_-currents in unmodified CHO cells intracellular solution contained (in mM): KCl 139.5, MgCl_2_ 3.5, DiBrBAPTA 2, HEPES 5, Na_2_ATP 2.5, Na_3_GTP 0.1 (pH 7.3); CaCl_2_ was added to obtain the following free [Ca^2+^]_pip_: 100 nM, 1 μM, 10 μM and 50 μM.

WEBMAXC STANDARD [https://somapp.ucdmc.ucdavis.edu/pharmacology/ /bers/maxchelator/webmaxc/webmaxcS.htm] was used for calculation of the appropriate amount of calcium to be added to the internal solution; final free Ca^2+^ concentrations were checked with a Ca^2+^-sensitive electrode (World Precision Instruments, Sarasota, USA). Steady-state activation of BK_Ca_ channels at distinct [Ca^2+^]_i_ was determined using test pulses ranging from −80 to +200 mV (in 10 mV increments), followed by a repolarization step to 0 or −50 mV. Conductance–voltage relations were determined from tail current amplitudes measured 0.5 ms after repolarization to the fixed membrane potential and normalized to maximum. Data were fitted with a Boltzmann function g/g_max_ = g_max_/(1+exp((V_h_-V_m_)/k)), where V_h_ is voltage required for half maximal activation and k is the slope factor.

BK_Ca_-currents in CHO cells stably expressing Cav2.2 channels were recorded using internal solution containing (in mM): KCl 127.5; MgCl_2_ 3.5; HEPES 5; Na_2_ATP 2.5; Na_3_GTP 0.1 and K_2_EGTA 0.1 oder 10 (pH 7.3). For determination of PMCA-mediated Ca^2+^-clearance via the deactivation time course of BK_Ca_ channels a previously established voltage-protocol^23^ was used. Briefly, a depolarizing voltage-step (from −90 to 70 mV) activated Cav2.2 (and a minor portion of BK_Ca_) channels without inducing Ca^2+^ influx (due to the lack of driving force); Ca^2^^+^ influx was triggered by a 0.8 ms voltage-step to −60 mV. The resulting increase in [Ca^2+^]_i_ activated BK_Ca_ channels and thus triggered transient (in the presence of PMCA and NPTN) or long-lasting (control) K^+^ currents that were measured at a potential of 70 mV. The current decay was fitted with a mono-exponential function yielding values for the respective time constant (τ _decay_). For the multiple calcium influx protocol (Fig. 1c, Fig. 4c) 2 or 5 repolarization pulses to −60 mV at 2 ms intervals (500 Hz) were applied between the pre- and the test-pulse. Data analysis and fitting was done using Igor Pro9 (WaveMetrics).

All chemicals except DiBrBAPTA (Alfa Aesar) were purchased from Sigma.

### Fluorescence microscopy

CHO cells were recorded in the whole-cell voltage-clamp mode using a EPC10 amplifier (Heka, Germany) under control of Pulse 8.5 (HEKA Electronic). Patch-pipettes had a resistance of 2–4 MΩ when filled with the intracellular solution described above. Cells with an average access resistance of 3–12 MΩ, with 50–75% resistance compensation and <20pA leak-current were used for analysis. Current signals were low-pass filtered at 2.9 kHz (four pole Bessel filter EPC10) and digitized at 50 kHz. Ca^2+^ signals were recorded simultaneously. Lck-GCaMP6s fluorescence was acquired with an Evolve EMCCD camera (Visitron, Germany) using a Zeiss Plan Apochromat 40x oil immersion objective (NA 1.3) on a Axiovert200 microscope (Zeiss). Fluorescent images were captured at 100 Hz with custom written macros in VisiView (Visitron, Germany), processed offline using ImageJ 1.43 software and Igor Pro5. Background was deducted by subtracting the F_0_ image (average of three pre-stimulus images) from all subsequent images (ΔF_n_ = F_n_-F_0_). Regions of interest of identical size (10×10 pixels) were placed over the plasma-membrane reacting to the electrical stimulation and fluorescence changes were tracked throughout the stack.

### Electron microscopy

#### Sample preparation, CHO cells

For determination of the density of Cav2.2, PMCA2 and NCX2 protein(s) in CHO cells the SDS-FRL technique was used as previously described^45^ with some modifications. CHO cells constitutively expressing Cav2.2 were transiently transfected with the aforementioned plasmids (coding for PMCA2-GFP, NPTN-RFP, NCX2-GFP) or an empty vector with Jet-PEI transfection reagent according to the manufacture’s instruction. After 2 days of incubation, cells were rinsed with 25 mM phosphate-buffered saline (PBS) and then fixed with 4% paraformaldehyde (Roth, Germany) in PBS for 10 min. Cells were harvested by scraping, pelleted by centrifugation (500 *g*, 5 min); cell pellets were sandwiched between copper carriers for high-pressure freezing (HPM100, Leica, Austria). Frozen pellets were then fractured into two parts at −120 °C and the fractured facets were coated with carbon (5 nm), platinum-carbon (2 nm) and an additional layer of carbon (20 nm) in a freeze-fracture replica machine (ACE900, Leica). Replicas were digested at 60 °C in a solution containing 2.5 % SDS and 20% sucrose diluted in 15 mM Tris buffer (pH 8.3) for 48 h followed by 37 °C for 20 h. The replicas were washed in washing buffer comprising 0.05 % bovine serum albumin (BSA, Roth, Germany) and 0.1% Tween20 (Tw20, Roth, Germany) in 50 mM Tris-buffered saline (TBS) and then blocked in a solution containing 5% BSA and 0.1% Tween20 in TBS at room temperature (RT) for 1 h. Subsequently, replicas were incubated at 15 °C two overnights (O/Ns) in the following mixtures of primary antibodies in a solution containing 1% BSA and 0.1% Tween20 made up in TBS: (i) PMCApan (5F10, mouse /Ms/, 1:3000, Invitrogen, Cat. MA3-914) and Cav2.2 (rabbit, 1.67 µg/ml, Synaptic Systems, Cat. 152303) or (ii) NCX2 (SLC8A2, 0.5 µg/ml, Alomone, Cat. ANX-012). Replicas were washed in washing buffer then reacted with 6 nm (Rb) or 12 nm (Ms or Rb) gold particle-conjugated secondary antibodies (1:60, Jackson ImmunoResearch Laboratories, PA) at 15 °C overnight.

#### Sample preparation, cerebellum

Immunogold labeling of replicas was performed as previously described^46^. Adult mice (*n* = 3; Charles River, Germany) were narcotized with isoflurane then anesthetized with pentobarbital (80 mg/kg). Cerebelli were removed and immersed into a fixative containing 2% paraformaldehyde and 15% saturated picric acid in 0.1 M phosphate buffer (PB) for 4 h at 4 °C. Cerebellar slices (100 µm) were cut on a vibrotome (VT 1000, Leica) and cryoprotected with 30% glycerol in 0.1 M PB O/N at 4 °C and then frozen by the high pressure freezing machine. Frozen samples were placed onto double replica tables then fractured at −140 °C and coated by deposition with carbon (5 nm), platinum (2 nm) and carbon (18 nm) in a freeze-fracture replica device (BAF 060 BAL-TEC, Liechtenstein). Replicas were digested at 80 °C for 18 h in a solution containing 2.5 % SDS and 20 % sucrose diluted in 15 mM TB. They were washed in 50 mM TBS containing 0.05% BSA and 0.1% Tween20 and then incubated in a blocking solution. Subsequently, replicas were incubated in the mixture of the following primary antibodies: anti-PMCA1-4 (PMCApan, Mouse, 1:3000, Invitrogen, Cat.# M;A3-914) and anti-Cav2.1 (Guinea pig, Gp, 5 μg/mL^47^,) prepared in 50 mM TBS containing 1% BSA and 0.1% Tween20 at RT. After washing them in TBS they were reacted with a mixture of 5 nm (Gp) and 10 nm (Ms) gold particle-conjugated secondary antibodies (1:50, BioCell Research Laboratories, Cardiff, UK) at 15 °C overnight.

#### Microscopy and quantification

Finally, replicas were washed in TBS followed by distilled water, mounted on Formvar-coated 100 mesh grids, and analyzed with transmission electron microscopes (CM100, Philips or Zeiss LEO 906 E). All antibodies targeted intracellular epitopes of proteins, immunoreactivity was therefore observed on the protoplasmic face (P-face) of the plasma membrane. The density of immuno-particles labeling protein(s) of interest was calculated by dividing the absolute number of gold particles by the surface area of CHO cells, dendritic shafts of Purkinje cells, and varicosities of parallel fibers. Data are given as individual data points and mean values ± SEM. Statistical significance was assessed by the non-parametric Mann-Whitney U-test.

### Biochemistry and proteomic analysis

#### Quantitative MS analysis on membrane fractions

Tissues from adult mice were freshly collected and snap-frozen before they were subjected to a simple total membrane isolation protocol^29^. 1 mg of membrane suspension was then extracted with 1 ml 100 mM Na_2_CO_3_ (pH 11) to reduce sample complexity. After ultracentrifugation, the pellets were solubilized with 100 µl ComplexioLyte91 (CL-91, Logopharm GmbH, Germany) and aliquots run on a short SDS-PAGE gel. After silver staining, the gel lanes were excised and subjected to standard tryptic in-gel digestion. Extracted and dried peptides were then dissolved in 0.5% (v/v) trifluoroacetic acid and loaded onto a precolumn (C18 PepMap100 (300 µm i.d. x 5 mm; particle size 5 µm, Thermo Scientific, Germany) for 5 min at 20 µL/min with 0.05% (v/v) trifluoroacetic acid using a split-free UltiMate 3000 RSLCnano HPLC (Thermo Scientific, Germany). Bound peptides were then eluted with an aqueous-organic gradient (eluent A: 0.5% (v/v) acetic acid; eluent B: 0.5% (v/v) acetic acid in 80% (v/v) acetonitrile): 5 min 3% B, 120 min from 3% B to 30% B, 20 min from 30% B to 50% B, 10 min from 50% B to 99% B, 5 min 99% B, 5 min from 99% B to 3% B, 15 min 3% B (flow rate 300 nl/min). Eluted peptides were separated in a PicoTip™ emitter (75 µm i.d.) manually packed 23.5 cm with ReproSil-Pur 120 ODS-3 (C18; particle size 3 µm; Dr. Maisch HPLC, Germany) and electrosprayed (2.3 kV; transfer capillary temperature 300 °C; funnel RF level 45.0) in positive ion mode into a LTQ Orbitrap XL tandem mass spectrometer (Thermo Scientific, Germany) with the instrument settings described in ref. ^29^.

For each dataset, a peak list was extracted from fragment ion spectra using the “msconvert.exe” tool (part of ProteoWizard; http:// proteowizard.sourceforge.net/; v3.0.11098; Mascot generic format with filter options “peakPicking true 1-” and “threshold count 500 most-intense”) and the precursor m/z values were shifted by the median m/z offset of all peptides assigned to proteins in a preliminary database search with 50 ppm peptide mass tolerance. Corrected peak lists were searched with Mascot 2.6.2 (Matrix Science, UK) against the UniProtKB/Swiss-Prot database (mouse, rat and human entries). Acetyl (Protein N-term), Carbamidomethyl (C), Gln->pyro-Glu (N-term Q), Glu->pyro-Glu (N-term E), Oxidation (M), Phospho (S, T, Y), and Propionamide (C) were chosen as variable modifications, peptide and fragment mass tolerance were set to ±5 ppm and ±20 mmu, respectively. One missed tryptic cleavage was allowed. The expect value cut-off for peptide assignment was set to 0.5. Related identified proteins (subset or species homologs) were grouped using the name of the predominant member. Proteins either representing exogenous contaminations (e.g., keratins, trypsin, IgG chains) or identified by only one specific peptide were not considered.

Label-free quantification of proteins was carried out following the principles and procedures described in refs. ^48–50^. Peptide signal intensities (peak volumes, PVs) from FT full scans were determined and offline mass calibrated using MaxQuant v1.6.3.3 (https://www.maxquant.org). Peptide PV elution times in evaluated datasets were pairwise aligned using LOESS regression (reference times were dynamically calculated from the median peptide elution times over all aligned datasets). PVs were then assigned to peptides based on their m/z and elution time obtained either directly from MS/MS-based identification or indirectly (i.e., from identifications in parallel datasets) using in-house developed software (matching tolerances of ±2 ppm and ±1 min). The obtained matrix of protein-specific peptide intensities / run was then subjected to consistency-based evaluation and used for weighted fitting to a global reference^49^. Molecular abundances of proteins (Fig. 2A and Supplementary Fig. 2) were estimated using the abundance_norm_spec measure^48^.

#### Cryo-slicing BN-MS

Synaptosome-enriched membranes were prepared from whole mouse brain^51^, solubilized with CL-47 (Logopharm GmbH, Germany; 1 ml/mg membrane protein, protease inhibitors added), and resolved on a preparative scale blue native polyacrylamide gradient (BN-PAGE) gel as described^50^. The section of interest was excised from the gel lane, embedded and cryo-sliced (stepsize 0,33 mm) as detailed in ref. ^52^. In-gel tryptic digestion and LC-MS/MS analysis were performed as follows: Peptides were dissolved in 0.5% (v/v) trifluoroacetic acid and loaded onto a precolumn (C18 PepMap100 (300 µm i.d. x 5 mm; particle size 5 µm, Thermo Scientific, Germany) for 5 min at 20 µL/min with 0.05% (v/v) trifluoroacetic acid using a split-free UltiMate 3000 RSLCnano HPLC (Thermo Scientific, Germany). Bound peptides were then eluted with an aqueous-organic gradient (eluent A: 0.5% (v/v) acetic acid; eluent B: 0.5% (v/v) acetic acid in 80% (v/v) acetonitrile): 5 min 3% B, 120 min from 3% B to 30% B, 20 min from 30% B to 50% B, 10 min from 50% B to 99% B, 5 min 99% B, 5 min from 99% B to 3% B, 10 min 3% B (flow rate 300 nl/min). Eluted peptides were separated in a SilicaTip™ emitter (75 µm i.d.) manually packed 23.5 cm with ReproSil-Pur 120 ODS-3 (C18; particle size 3 µm; Dr. Maisch HPLC, Germany) and electrosprayed (2.3 kV; transfer capillary temperature 300 °C; funnel RF level 45.0) in positive ion mode into a Q Exactive HF-X mass spectrometer (Thermo Scientific, Germany). Full MS scan range was 370–1700 m/z with a target value of 3*10^6 ^ions at a nominal resolution of 240,000. Each precursor scan was followed by up to 15 data-dependent MS/MS fragmentation spectra with a target value of 10^5 ^ions (min. 8*1^03^ IT_max_ = 200 ms), isolation window 1.0 m/z, resolution of 15,000. Isotopes and charge states +1, >+8 were skipped, dynamic exclusion was set to 60 s.

LC-MS (PV) data were extracted and assigned to peptides as described above and then further processed following the steps outlined in ref. ^52^: Slice-to-slice sample variations in peptide recovery and ionization efficiency of the LC-MS setup were corrected for by calculating the median PV offset of each slice from its predecessor and subsequent PV rescaling of each dataset by the median of all offset values inside a window of ±20 slices. Resulting PV data for each protein were filtered for outliers and false-positive assignments as identified by a correlation-based consistency check. Protein abundance distribution profiles were calculated from the resulting PV matrix by fitting the data to a globally determined abundance_norm_spec reference for each individual protein. Slice numbers were finally converted to apparent complex molecular weights by linear regression (log10(MW) versus slice number of the respective protein profile peak maximum) of the marker protein complexes (from the mitochondrial oxidative respiratory chain) described by Schägger and Pfeiffer^53^.

### Computational modelling

BK_Ca_-currents in response to voltage-changes at constant [Ca^2+^]_i_ (activation curves under steady-state conditions as in Fig. 1b), as well as to time-dependent changes in [Ca^2+^]_i_ at constant voltage(s) ([Ca^2+^]_i_ pulse-experiments as in Fig. 1c) were modelled in a spherical 3D-reaction-diffusion model with dimensions of an average CHO cell (‘model cell’) equipped with PMCA-NPTN complexes and Cav channels at the experimentally derived densities; 30% of the intracellular volume represents freely diffusible space, while 70% are inaccessible to free diffusion (all values and parameters summarized in Supplementary Table 1).

#### Spatio-temporal profiles for intracellular Ca^2+^ concentration

Under *steady-state conditions* (as in the experiments of Figs. 1b and 4a), free Ca^2+^ in the diffusion-accessible volume obeys the stationary diffusion equation ${{D}_{c}\nabla }^{2}c\left(\vec{x}\right)=0$ with influx and outflux of Ca^2+^ defined as follows (see also Supplementary Table 1): Dirichlet boundary conditions at the pipette-cell contact area (∂Ω_pip_) $c\left(\vec{x}\right){|}_{\vec{x}\epsilon \partial \Omega {{{\rm{pip}}}}}={c}_{{pip}}$, and von Neumann boundary conditions at the cell surface (∂Ω_PMCA_) outside the pipette-covered area

1 $${D}_{c}{\nabla }_{{{{\boldsymbol{n}}}}}c\left(\vec{x}\right){|}_{\vec{x}\epsilon \partial \Omega {{{\rm{pmca}}}}}={\vec{{{{\boldsymbol{j}}}}}}_{{PMCA}}(\vec{x})+{\vec{{{{\boldsymbol{j}}}}}}_{{leak}}$$

where ${\vec{{{{\boldsymbol{j}}}}}}_{{PMCA}}$ is the Ca^2+^ current by the PMCA2-NPTN-mediated transport given as

2 $${\vec{{{{\boldsymbol{j}}}}}}_{{PMCA}}\left(\vec{x}\right)={{\phi }}_{{PMCA}}{{\rho }}_{{PMCA}}f(c\left(\vec{x}\right))$$

and ${\vec{{{{\boldsymbol{j}}}}}}_{{leak}}$ is a constant Ca^2+^ leak current. In Eq. 2, ${\phi }_{{PMCA}}$ denotes the cycle rate of the PMCA pumps, ${\rho }_{{PMCA}}$ is their experimentally determined density of 50/µm^2^ (Fig. 2) and $f(c)$ describes the concentration dependence of PMCA transport-activity; $f(c)$ is a Hill-function of the local Ca^2+^ concentration $c$ at the membrane with values for c_1/2_ and Hill-coefficient of 0.43 µM and 2, respectively^54^. Setting ${\vec{{{{\boldsymbol{j}}}}}}_{{leak}}={\vec{{{{\boldsymbol{j}}}}}}_{{PMCA}}({c}_{0})$ fixed the net flux of Ca^2+^ to zero at the resting Ca^2+^ concentration ${c}_{0}$ of 0.1 µM. For determination of spatial [Ca^2+^]_i_ profiles in the diffusion-accessible intracellular volume under steady-state conditions with [Ca^2+^]_pipette_ of 10 µM and variable values for ${\phi }_{{PMCA}}$, the stationary diffusion equation with boundary conditions (Eqs. 1, 2) was numerically solved using FreeFEM + +^55^ (Fig. 3a).

Under the *dynamic conditions* of the ‘pulsed Ca^2+^-experiments’ (Figs. 1c, f, 4c), the time-course of [Ca^2+^]_i_ after the 0.8 ms-influx pulse is modeled by reaction-diffusion equations for Ca^2+^ and buffer (EGTA) concentration, $c\left(t,\vec{x}\right)$ and $b\left(t,\vec{x}\right)$, respectively:

3 $$\frac{\partial c\left(t,\vec{x}\right)}{\partial t}={D}_{c}{\nabla }^{2}c\left(t,\vec{x}\right)-{k}_{+}c\left(t,\vec{x}\right)b\left(t,\vec{x}\right)+{k}_{-}\left({{{{\rm{b}}}}}_{{tot}}-b\left(t,\vec{x}\right)\right)$$

4 $$\frac{\partial b\left(t,\vec{x}\right)}{\partial t}={D}_{b}{\nabla }^{2}b\left(t,\vec{x}\right)-{k}_{+}c\left(t,\vec{x}\right)b\left(t,\vec{x}\right)+{k}_{-}\left({{{{\rm{b}}}}}_{{tot}}-b\left(t,\vec{x}\right)\right)$$

${D}_{c}$ and ${D}_{b}$ are the diffusion constants for Ca^2+^ and EGTA, respectively, ${k}_{+}$ and ${k}_{-}$ the binding and unbinding rates of Ca^2+^ to EGTA^32^, and b_tot_ the total EGTA concentration (0 or 10 mM, all parameters in Supplementary Table 1). Ca^2+^ influx and outflux are described by von Neumann boundary conditions

5 $${D}_{c}{\nabla }_{{{{\boldsymbol{n}}}}}c\left(t,\vec{x}\right){|}_{\vec{x}\epsilon \partial \Omega {{{\rm{pmca}}}}}={\vec{{{{\boldsymbol{j}}}}}}_{{PMCA}}+{\vec{{{{\boldsymbol{j}}}}}}_{{leak}}+{\vec{{{{\boldsymbol{j}}}}}}_{{CaV}}$$

where ${\vec{{{{\boldsymbol{j}}}}}}_{{CaV}}$ is Cav2-mediated Ca^2+^ current (during the 0.8 voltage-pulse), ${\vec{{{{\boldsymbol{j}}}}}}_{{PMCA}}$ and ${\vec{{{{\boldsymbol{j}}}}}}_{{leak}}$ are as in Eqs. 1, 2. ${\vec{{{{\boldsymbol{j}}}}}}_{{CaV}}$ is experimentally determined average number of Ca^2+^ ions entering the cell during the 0.8 ms-pulse (1.2 ∙ 10^6^ / 0.8 ms) divided by the cell surface (300 µm^2^), and zero otherwise. The accessible intracellular volume (30% of total) resulted in a [Ca^2+^]_i_ at the end of the 0.8 ms-influx pulse of around 10 µM, a minimal value to account for the observed rapid onset of the Ca^2+^-activated BK_Ca_ currents^23^.

For determination of the spatio-temporal evolution of [Ca^2+^]_i_ following the 0.8 ms-influx pulse with or without buffer, with variable values for ${\phi }_{{PMCA}}$, the above formulated reaction diffusion problem (Eqs. 3–5) was numerically solved using FreeFEM + +.

#### Gating model of BK_Ca_ channels

For theoretical approximation of measured BK_Ca_-currents (Figs. 1, 4) we used a previously established model for BK_Ca_ channel gating^30^. Briefly, this model comprises a total of 10 distinct states, five open (O _0-4_) and five closed (C _0-4_) states, and four binding sites for Ca^2+^ ions (occupancy designated 0-4). Transitions between states O_i_ and C_i_ (i = 0,…, 4) are voltage-dependent, while transitions between states O_i_ and O_i+1_ and C_i_ and C_i+1_ (i = 0,…, 3) are dependent on [Ca^2+^]_i_ (Supplementary Tables 2, 3).

For steady-state conditions, the open probability of the model (P_open_) is given by

6 $${P}_{{open}}={\left(1+B(c)\cdot L(0)\cdot {e}^{-\frac{{QFV}}{{RT}}}\right)}^{-1}$$

where V denotes membrane voltage, Q is elementary charge, and R, T and F having their usual meaning; L(0) is open to close equilibrium constant at 0 mV with no Ca^2+^ bound. The Ca^2+^ concentration dependent factor $B\left(c\right)$ is given by

7 $$B\left(c\right)=\left(\frac{{c}^{4}}{{K}_{C1}{K}_{C2}{K}_{C3}{K}_{C4}}+\frac{{c}^{3}}{{K}_{C1}{K}_{C2}{K}_{C3}}+\frac{{c}^{2}}{{K}_{C1}{K}_{C2}}+\frac{c}{{K}_{C1}}+1\right)/ \\ \left(\frac{{c}^{4}}{{K}_{O1}{K}_{O2}{K}_{O3}{K}_{O4}}+\frac{{c}^{3}}{{K}_{O1}{K}_{O2}{K}_{O3}}+\frac{{c}^{2}}{{K}_{O1}{K}_{O2}}+\frac{c}{{K}_{O1}}+1\right)$$

K_Ci_ and K_Oi_ are the dissociation constants for Ca^2+^ binding to closed and open states of the BK_Ca_ channels, respectively.

This gating model was calibrated with experimentally determined BK_Ca_-activation curves (as in Fig. 1b) for [Ca^2+^]_i_ values of 0.1, 1, 10, 50 µM^10^ via an optimization routine^56^. The respective values obtained for L(0), Q and the reaction constants K_Ci_ and K_Oi_ (detailed in Supplementary Table 2) were finally used together with the spatial intracellular [Ca^2+^]_i_ profiles, $c\left(\vec{x}\right)$, to calculate the open probability ${P}_{{open}}\left(c(\vec{x})\right)$ of the channels distributed over the entire surface of the model cell. Averages of these ${P}_{{open}}\left(c(\vec{x})\right)$ provided the activation curves under steady-state conditions with a fixed [Ca^2+^]_pipette_ of 10 µM and variable values for ${\phi }_{{PMCA}}$ (Fig. 3a).

For modelling BK_Ca_-responses to the *dynamic conditions* given in the ‘Ca^2+^-pulse experiments’ (as in Fig. 1c), the channel model was extended to include forward and backward rates (Supplementary Table 3). The open probability P_open_ (t) is given as

8 $${P}_{{open}}\left(t\right)=\frac{{O}_{{tot}}(t)}{{C}_{{tot}}\left(t\right)+{O}_{{tot}}(t)}=\frac{1}{1+{\sum }_{i=0}^{4}{C}_{i}(t)/{\sum }_{i=0}^{4}{O}_{i}(t)}$$

The extended model was calibrated with experimental data previously obtained on BK_Ca_ channels exposed to sub-millisecond switches of [Ca^2+^]_i_ under constant membrane potential conditions (^23^; Supplementary Fig. 5); the respective values for Q and the reaction constants fitting the experimental data best are summarized in Supplementary Table 3.

Again, these parameters were used together with the time-dependent intracellular [Ca^2+^]_i_ profiles, $c\left(\vec{x},t\right)$, to compute the time course of ${P}_{{open}}\left(t\right)$ that provided the response(s) of the BK_Ca_-model to the 0.8 ms-pulses of [Ca^2+^]_I_ under defined values of ${\phi }_{{PMCA}}$ (Fig. 3b, Supplementary Fig. 5c).

### Quantification and data analysis

All statistical details are indicated in the figure legends. Data are given as mean ± SEM throughout the manuscript and are analyzed as detailed in the Method section. Significance was assessed by the non-parametric Mann-Whitney U-test, significance levels are indicated (***, **, * for *p* values < 0.001, 0.01, 0.05, respectively; n.s. is not significant).

### Reporting summary

Further information on research design is available in the Nature Portfolio Reporting Summary linked to this article.

## Supplementary information

Supplementary Information

Reporting Summary

Transparent Peer Review file

## Source data

Source data

## Data Availability

The mass spectrometry data (Fig. 2) have been deposited to the ProteomeXchange Consortium via the PRIDE partner repository with the dataset identifier PXD060109 [10.6019/PXD060109]. Source data are provided with this paper.
