# Supplementary material for: Ca2+-pumping by PMCA-neuroplastin complexes operates in the kiloHertz-range
Source: Nat Commun. 2025 Aug 20;16:7550. doi: 10.1038/s41467-025-62735-5 (PMC12368035; doi:10.1038/s41467-025-62735-5)
Supplement: Supplementary file 1 — Supplementary Information [file 41467_2025_62735_MOESM1_ESM.pdf]

Supplementary Material

Ca^{2+}-pumping by PMCA-Neuroplastin complexes operates in the kiloHertz-range

Cristina E. Constantin, Barbara Schmidt, Yvonne Schwarz, Harumi Harada, Astrid Kollewe, Catrin S. Müller, Sebastian Henrich, Botond Gaal, Akos Kulik, Dieter Bruns, Uwe Schulte, Heiko Rieger and Bernd Fakler

- Supplementary Figures 1 - 5
- Supplementary Tables 1 - 3

Supplementary Figures

Supplementary Figure 1 (information related to Figure 1)

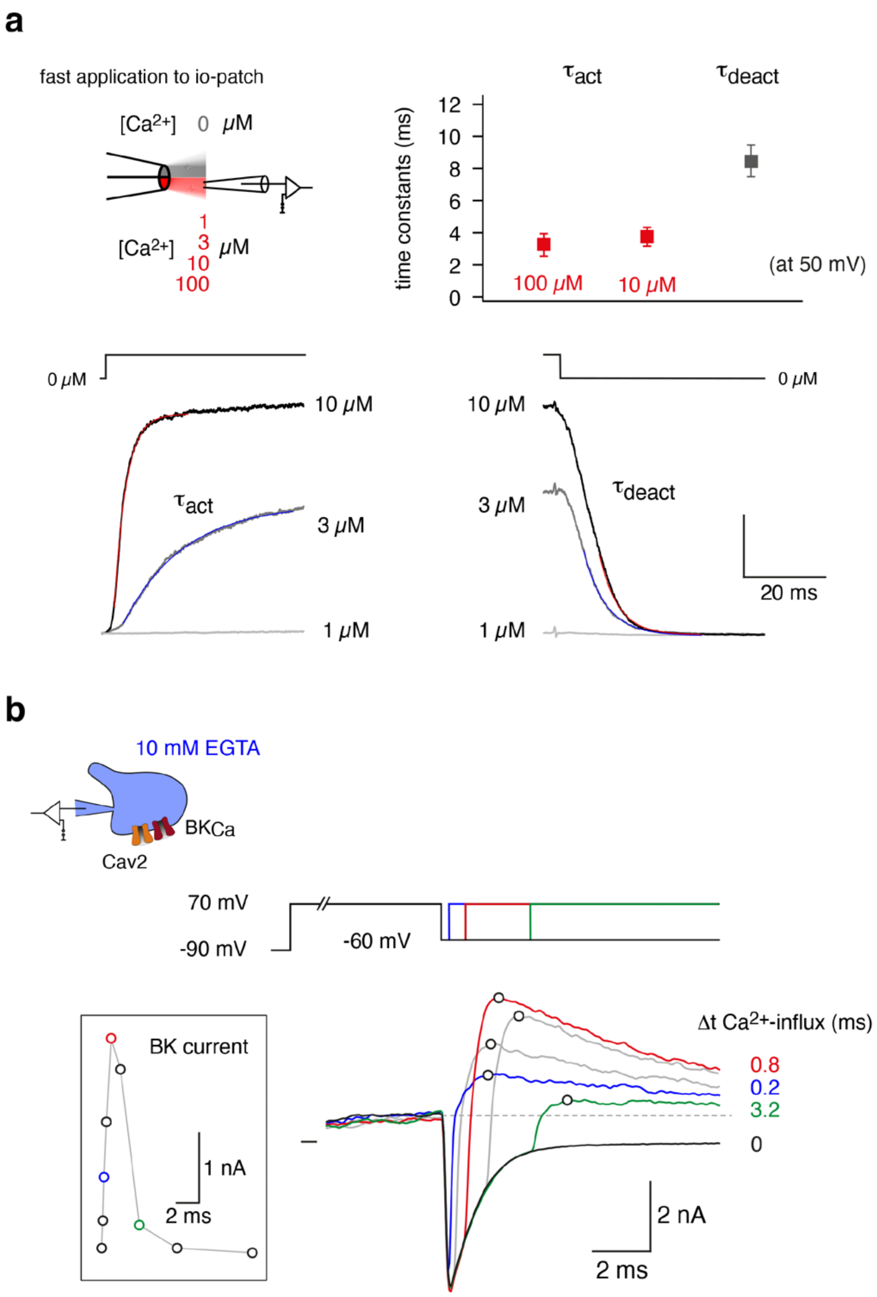

Supplementary Figure 1

Ca^{2+}-gating of BK_{Ca} channels

a, Activation and deactivation of BK_{Ca} channels determined at a membrane potential of 50 mV with a piezo-driven fast-application system exchanging $[Ca^{2+}]_i$ at the cytoplasmic face of the channels in the sub-millisecond range (termed: Ca^{2+}-gating [23]). Upper left panel: Scheme of the fast-application system exchanging solutions with the indicated values for $[Ca^{2+}]_i$ and a Ca^{2+}-free solution. Upper right panel: Values for activation and deactivation time constants of Ca^{2+}-gating at 50 mV. Lower panel: Representative current responses of BK_{Ca} channels to either an increase in $[Ca^{2+}]_i$

(from 0 to 1, 3 or 10 μM (left) or a decrease in $[Ca^{2+}]_i$ (from 1, 3 and 10 μM to 0 (right)). Lines represent fits of a mono-exponential function to the activation and deactivation phases with time constants of 2.8 ms (red) and 14.7 ms (blue) for activation at 10 and 3 μM Ca^{2+}, respectively, and 6.0 ms (red) and 5.3 ms (blue) for deactivation from 10 and 3 μM Ca^{2+}, respectively. Time scale as indicated, current scale is 0.5 nA. (Data were taken from [23]).

b, Current responses (color-coded) recorded in a CHO cell with the indicated voltage-protocol, protein expression and ion conditions were identical to the experiments in Fig. 1c, the patch pipette contained 10 mM EGTA. Trace in black reflects the Ca^{2+}-inward current through Cav2.2 channels (following step-repolarization from 70 mV to -60 mV), while colored traces highlight BK_{Ca}-mediated outward K^+ currents as in Fig. 1c activated by Ca^{2+}-influx of 0.2 ms (blue), 0.8 ms (red) and 3.2 ms (green) duration, as well as by Ca^{2+}-influx of 0.4 and 1.6 ms (light grey, for better discrimination).

Note the accurate monitoring of $[Ca^{2+}]_i$ by the BK_{Ca}-current maximum. Scaling for time and current as indicated, dashed line denotes BK_{Ca}-current prior to Ca^{2+}-influx, small line is zero current.

Supplementary Figure 2 (information related to Figure 1)

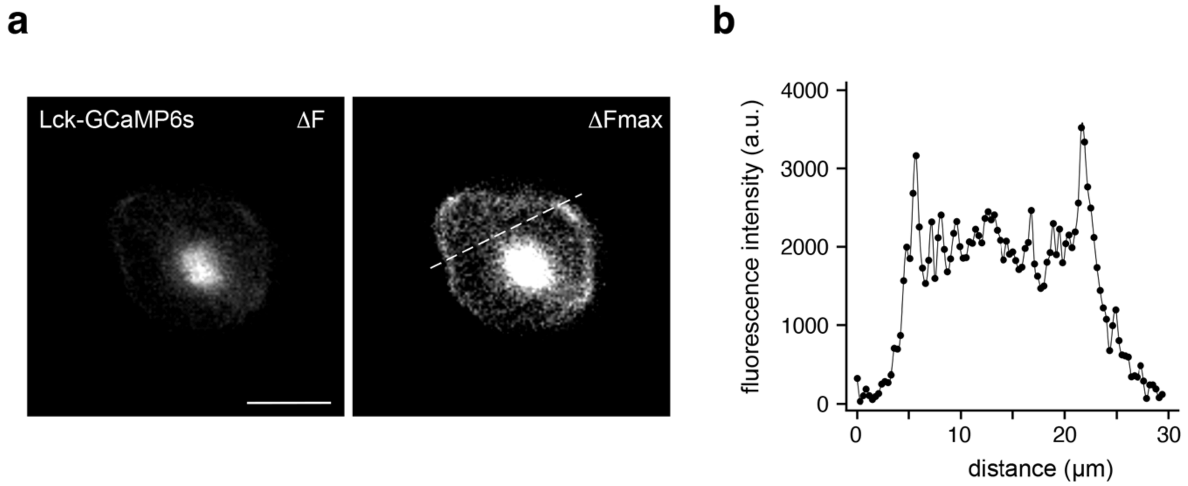

Supplementary Figure 2

Fluorescence measurements by membrane-tethered GCaM6s

a, Representative image of a CHO cell expressing Lck-GCaM6s before (left panel) and during/after step stimulation (right panel) with the voltage protocol in Fig. 1e. Dashed line is line-scan used for fluorescence measurement. **b**, Fluorescence intensity obtained along the line in (a).

Supplementary Figure 3 (information related to Figure 1)

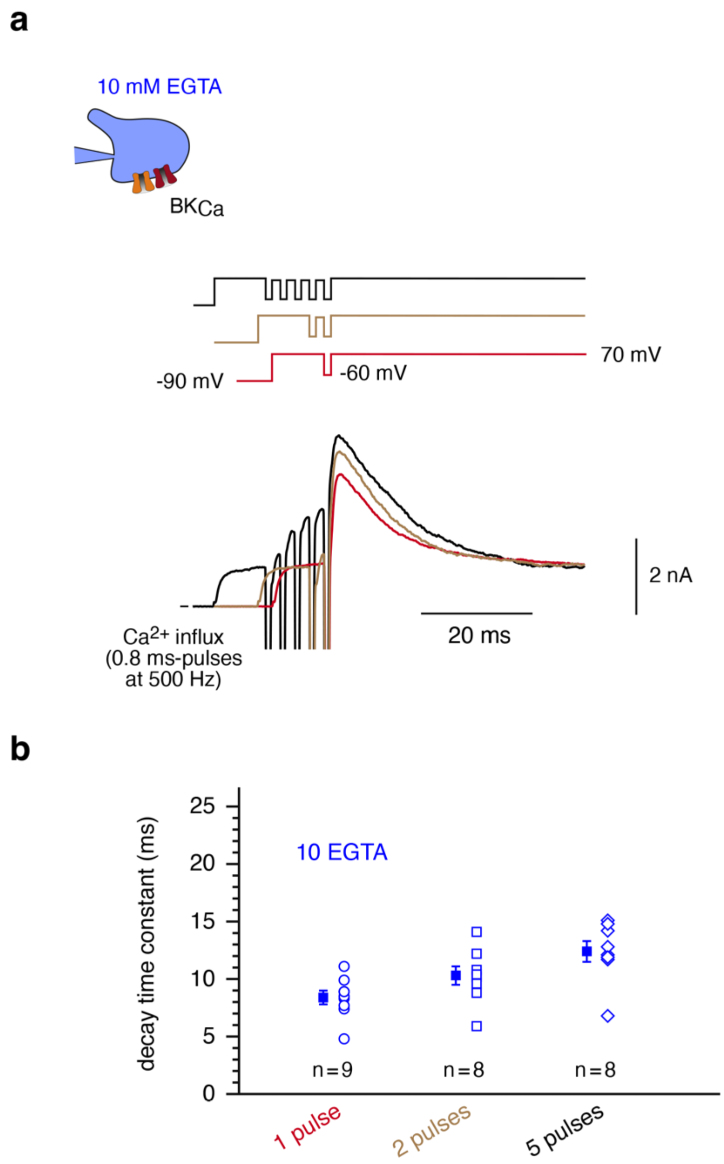

Supplementary Figure 3

Ca^{2+}-clearance by 10 mM EGTA in pulse-experiments

a, Representative BK_{Ca}-currents recorded in response to 1, 2 and 5 Ca^{2+}-influx pulses applied at 500 Hz (voltage protocol in lower inset) to CHO cells as in Figure 1, but with 10 mM EGTA in the patch-pipette (upper inset). **b**, Plot summarizing the time constants of the current decay determined in experiments as in (**a**); squares represent mean ± SEM of the indicated number of cells.

Supplementary Figure 4 (information related to Figure 2)

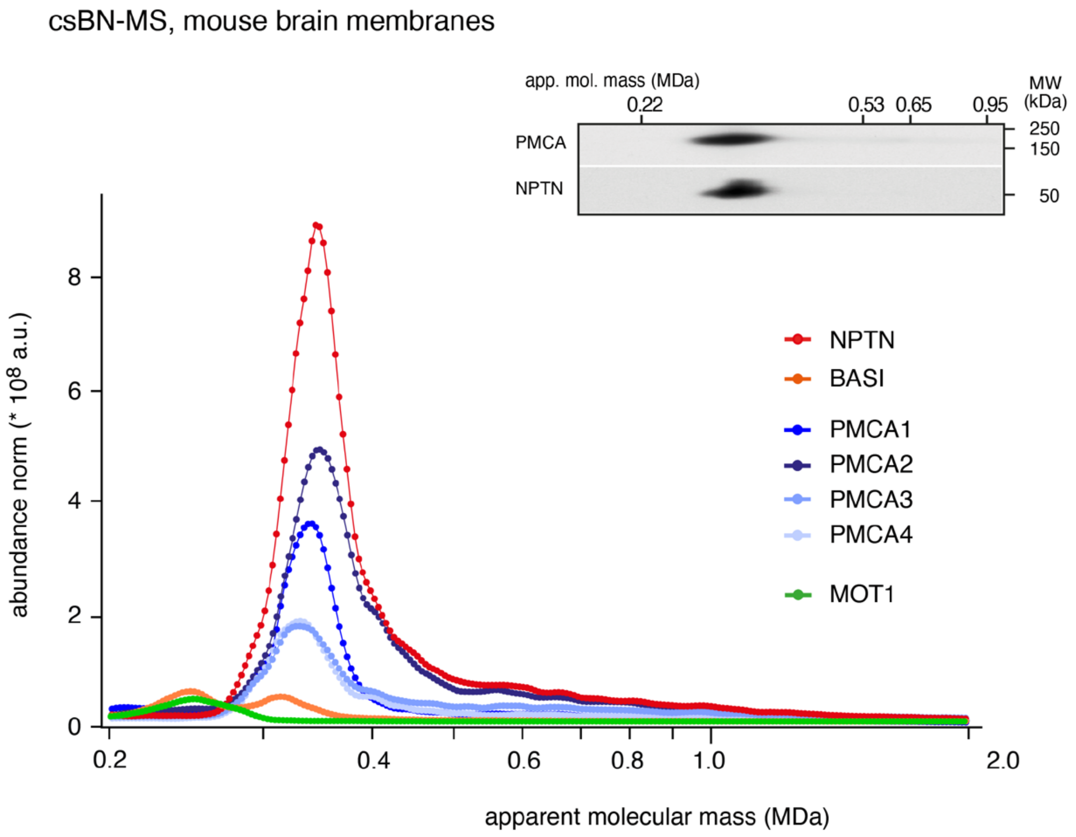

Supplementary Figure 4

Abundance-mass profiles of the constituents of native PMCA complexes in the mouse brain

Abundance-mass profiles obtained by cryo-slicing BN-MS (csBN-MS, [50]) for the indicated proteins in a CL-47 solubilized membrane fractions from adult mouse brain (a total of 170 gel slices). Note the co-segregation of all PMCA isoforms with NPTN and a sub-population of BASI. The other sub-population of BASI co-assembles with the mono-carboxylate-transporter MOT1. Inset: Two-dimensional gel separation of PMCA1-4 and NPTN in the same membrane fractions, Western-probed with antibodies targeting PMCA1-4 and NPTN. Size (BN-PAGE) and molecular weight (SDS-PAGE) are as indicated.

Supplementary Figure 5 (information related to Figure 3)

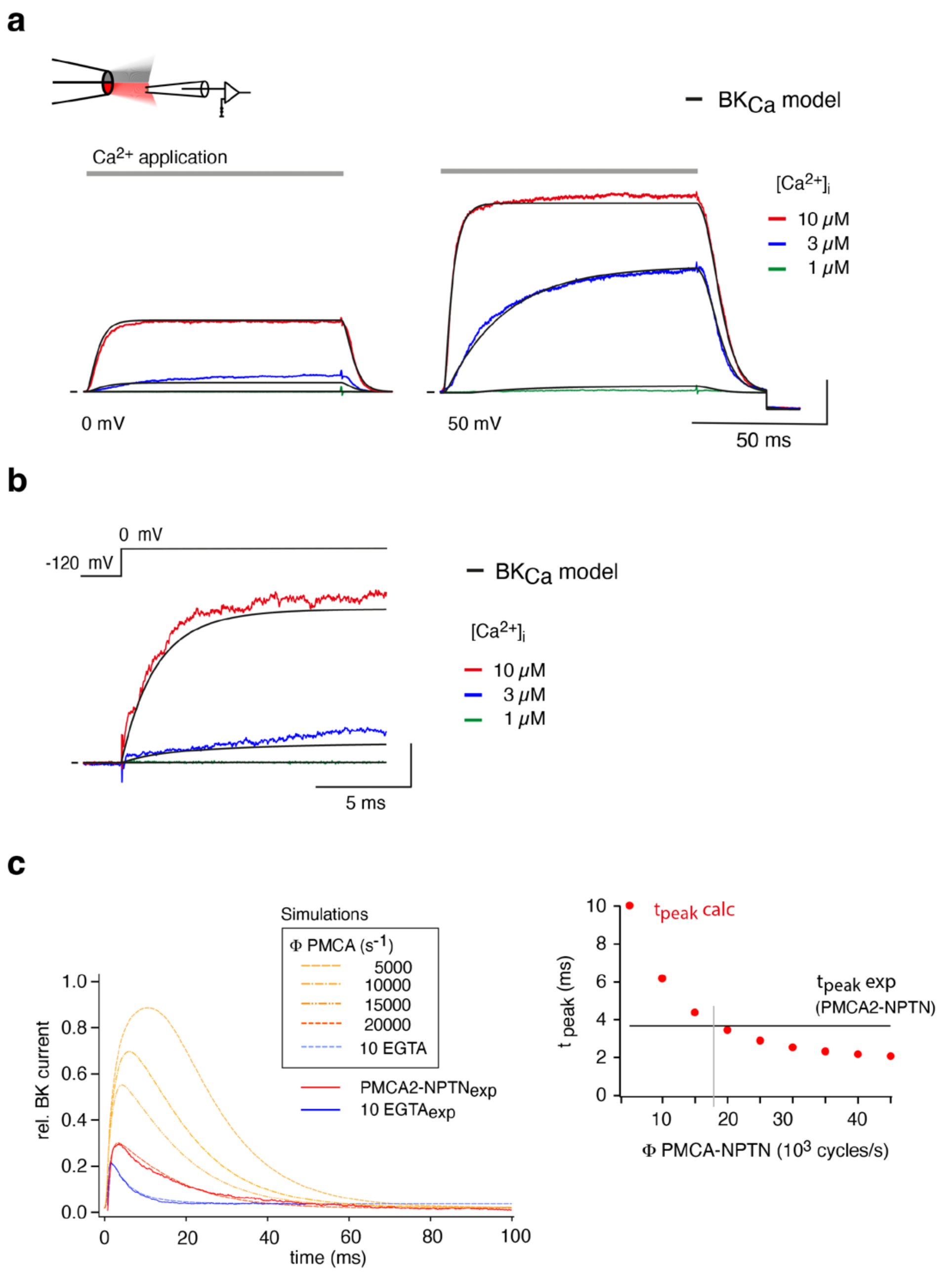

Supplementary Figure 5

Calibration of the gating model of BK_{Ca} channels

a, b Calibration of the extended BK_{Ca}-model via parameter-fitting to the BK_{Ca}-mediated currents recorded in experiments as in Extended Data Figure 1 (data taken from [23]). BK_{Ca}-currents were elicited by fast application of solutions with the indicated $[Ca^{2+}]_i$ at the indicated constant membrane potential (**a**, Ca^{2+}-gating), or by the indicated voltage-step with the indicated $[Ca^{2+}]_i$ constantly present at the cytoplasmic face of the channels (**b**, voltage-gating). Black lines represent the response of the BK_{Ca}-model to the respective changes in $[Ca^{2+}]_i$ and membrane potential. Note the good approximation of the experimental data by the calibrated model (parameters of the gating model are given in Supplementary Table 3).

c, Dependence of the time-to-peak (t_{peak}) of the BK_{Ca}-current maximum on the transport rate of the PMCA2-NPTN pumps (right panel) obtained from the calculations in Figure 3 (left panel). Note that perfect match between experimentally measured t_{peak} (black line, t_{peak} exp (right panel) or current trace in red (left panel)) and calculated t_{peak} (t_{peak} calc) is observed at a transport rate of about 18.000/s (grey line, right panel).

Supplementary Tables

Supplementary Table 1.

Parameters defining model cell and spatio-temporal profiles for intracellular Ca^{2+} concentration.

parameter	value	description
c_0	0.1 µM [1]	$[Ca^{2+}]$ at rest
D_c	0.22 $µm^2$/ms [1]	Ca^{2+} diffusion constant
r	5 µm	cell radius
r_{pip}	1.25 µm	pipette radius
c_{pip}	10 µM	$[Ca^{2+}]$ at pipette
ρ_{PMCA}	50/$µm^2$ [2]	surface density of PMCA-NTPN
$c_{1/2}$	0.43 µM [3]	Hill parameter
n	2 [3]	Hill coefficient
Φ_{PMCA}	free parameter	PMCA pump strength
b_t	0 or 10 mM [2]	total amount of [EGTA]
D_b	0.113$µm^2$/ms	EGTA diffusion constant
b_{pip}	0 or 10 mM [2]	[EGTA] at pipette
k_+	2.55 /µMs [1]	on-rate for Ca^{2+}-EGTA binding
k_-	0.45/s [1]	off-rate for Ca^{2+}-EGTA binding
N_{CaV}	1.200 000 [2]	number of incoming Ca^{2+}
Δt	0.8 ms [2]	duration of the voltage pulse

References

[1] citation 32
[2] this study
[3] citation 54

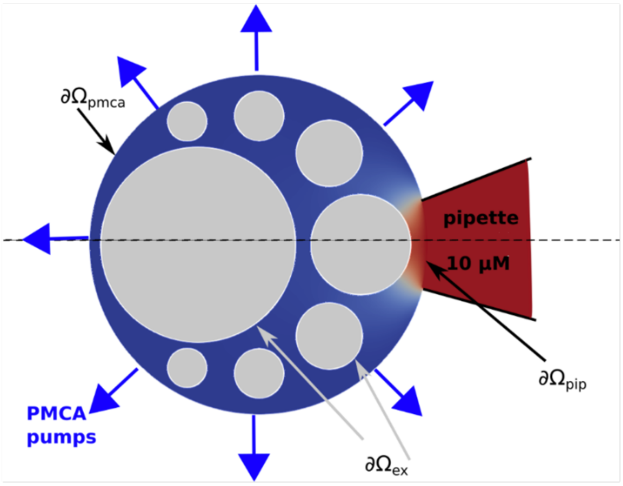

Equatorial cross-section of the spherically shaped model geometry
The three-dimensional geometry is generated by rotating this cross section around its symmetry axis indicated by the straight dashed line. The outer boundary ∂Ω of the cell volume Ω is partitioned into ∂Ωpmca comprising PMCA, Cav and leak Ca^{2+} currents, and ∂Ωpip with pipette solution-defined fixed Ca^{2+} concentration. Grey regions denote cross sections of intracellular organelles whose volumes are inaccessible to diffusing Ca^{2+} (70% of total intracellular volume), the boundaries, ∂Ωex, of these excluded sub-volumes have no-flux boundary conditions for Ca^{2+} diffusion.

Supplementary Table 2.

Parameters defining the BK_{Ca}-gating model used for computing the response to stationary conditions in $[Ca^{2+}]_i$.

parameter	value
Q	0.94 e
K_{C1}	27.1 µM
K_{C2}	0.09 µM
K_{C3}	8.57 µM
K_{C4}	7.74 µM
L(0)	80.5
K_{O1}	15.08 µM
K_{O2}	0.06 µM
K_{O3}	0.6 µM
K_{O4}	4.29 µM

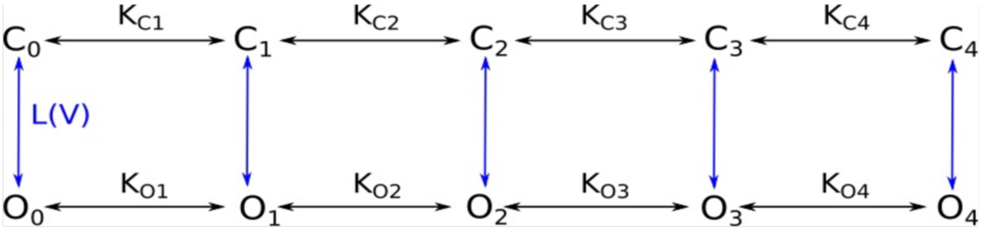

Stationary gating scheme for BK_{Ca}, channels (adapted and modified from (30)).
Voltage dependent rates L(V) = L(0)•exp(-QFV/RT) define the transition between open and closed conformation, where L(0) is the open-to-closed equilibrium constant in the absence of bound Ca^{2+} at 0 mV, V is transmembrane voltage, Q is equivalent gating charges associated with the closed-to-open conformational change and R, T, F have their usual meaning. K_{Ci} and K_{Oi} define binding of Ca^{2+} to the four binding sites of BK_{Ca} (used in eq. 7)

Supplementary Table 3.

Parameters defining the extended BK_{Ca}-gating model used for computing the response to pulsed increases in $[Ca^{2+}]_i$.

parameter	value	parameter	value
γ_0^+	1/18.03 s	γ_0^-	1/0.0009 s
γ_1^+	1/80.02 s	γ_1^-	1/0.016 s
γ_2^+	1/0.98 s	γ_2^-	1/0.0012 s
γ_3^+	1/0.008 s	γ_3^-	1/0.00016 s
γ_4^+	1/0.006 s	γ_4^-	1/0.002 s
Q_1	2.25e	Q_2	-0.11e
k_{C1}^+	34.1/µMs	k_{C1}^-	1741.68/s
k_{C2}^+	3972.8/µMs	k_{C2}^-	1015.79/s
k_{C3}^+	208.92/µMs	k_{C3}^-	20617.6/s
k_{C4}^+	711.2/µMs	k_{C4}^-	4449.017/s
K_{O1}^+	537.19/µMs	K_{O1}^-	7536.7/s
K_{O2}^+	727.16/µMs	K_{O2}^-	29.81/s
K_{O3}^+	32.63/µMs	K_{O3}^-	20.9/s
K_{O4}^+	314.8/µMs	K_{O4}^-	121.1/s

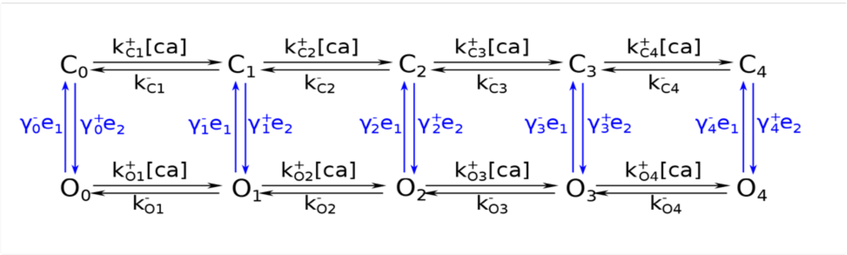

Gating scheme for BKCa - non stationary case (adapted and modified from (30)).
Voltage-dependent transition rates are defined as $e_1(V) = exp•(Q_1FV/RT)$ and $e_2(V) = exp•(Q_2 FV/RT)$, with V being transmembrane voltage and R, T, F and Q_i as in Supplementary Table 2.
