# Supplementary material for: Ca2+-pumping by PMCA-neuroplastin complexes operates in the kiloHertz-range
Source: Nat Commun. 2025 Aug 20;16:7550. doi: 10.1038/s41467-025-62735-5 (PMC12368035; doi:10.1038/s41467-025-62735-5)
Supplement: Supplementary file 2 — Reporting Summary [file 41467_2025_62735_MOESM2_ESM.pdf]

nature portfolio

Corresponding author(s): Bernd Fakler, Heiko Rieger

Last updated by author(s): July 24, 2025

Reporting Summary

Nature Portfolio wishes to improve the reproducibility of the work that we publish. This form provides structure for consistency and transparency in reporting. For further information on Nature Portfolio policies, see our Editorial Policies and the Editorial Policy Checklist.

Statistics

For all statistical analyses, confirm that the following items are present in the figure legend, table legend, main text, or Methods section.

n/a	Confirmed	
	x	The exact sample size (*n*) for each experimental group/condition, given as a discrete number and unit of measurement
	x	A statement on whether measurements were taken from distinct samples or whether the same sample was measured repeatedly
	x	The statistical test(s) used AND whether they are one- or two-sided<br>*Only common tests should be described solely by name; describe more complex techniques in the Methods section.*
x		A description of all covariates tested
x		A description of any assumptions or corrections, such as tests of normality and adjustment for multiple comparisons
	x	A full description of the statistical parameters including central tendency (e.g. means) or other basic estimates (e.g. regression coefficient) AND variation (e.g. standard deviation) or associated estimates of uncertainty (e.g. confidence intervals)
	x	For null hypothesis testing, the test statistic (e.g. *F*, *t*, *r*) with confidence intervals, effect sizes, degrees of freedom and *P* value noted<br>*Give P values as exact values whenever suitable.*
x		For Bayesian analysis, information on the choice of priors and Markov chain Monte Carlo settings
x		For hierarchical and complex designs, identification of the appropriate level for tests and full reporting of outcomes
x		Estimates of effect sizes (e.g. Cohen's *d*, Pearson's *r*), indicating how they were calculated

Our web collection on statistics for biologists contains articles on many of the points above.

Software and code

Policy information about availability of computer code

Data collection	The individual datasets were acquired with the following commercially available software (in combination with respective hardware)<br>msconvert v3.0.11098 for processing of primary MS data (obtained with a LTQ Orbizrap XL mass spectrometer)<br>Pulse 8.5 software (combined with a HEKA EPC10 amplifier for electrophysiology)<br>VisiView (combined with anEvolve EMCCD camera (Visitron) on a Axiovert 200 microscop (Zeiss, Plan Apochromat 40x) for fluorescence recording)<br>Built-in software of TEM microscopes (CM100 Philips or Zeiss LEO 906 E) for EM
Data analysis	msconvert v3.0.11098 for processing of primary MS data<br>MaxQuant 1.6.17 for callibration and quantification of MS data<br>Mascot 2.7 for database search<br>HEKA Pulse 8.5 and IGOR Pro 9 for evaluation of electrophysiological data<br>ImageJ 1.43 for analysis of fluoresecence images<br>FreeFEM++ for computational modelling

For manuscripts utilizing custom algorithms or software that are central to the research but not yet described in published literature, software must be made available to editors and reviewers. We strongly encourage code deposition in a community repository (e.g. GitHub). See the Nature Portfolio guidelines for submitting code & software for further information.

Data

Policy information about availability of data

All manuscripts must include a data availability statement. This statement should provide the following information, where applicable:

- Accession codes, unique identifiers, or web links for publicly available datasets
- A description of any restrictions on data availability
- For clinical datasets or third party data, please ensure that the statement adheres to our policy

The mass spectrometry data (Figure 2) have been deposited to the ProteomeXchange Consortium via the PRIDE partner repository with the dataset identifier PXD060109 and 10.6019/PXD060109, and are publicly available as of the date of publication.

Research involving human participants, their data, or biological material

Policy information about studies with human participants or human data. See also policy information about sex, gender (identity/presentation), and sexual orientation and race, ethnicity and racism.

Reporting on sex and gender	*Use the terms sex (biological attribute) and gender (shaped by social and cultural circumstances) carefully in order to avoid confusing both terms. Indicate if findings apply to only one sex or gender; describe whether sex and gender were considered in study design; whether sex and/or gender was determined based on self-reporting or assigned and methods used. Provide in the source data disaggregated sex and gender data, where this information has been collected, and if consent has been obtained for sharing of individual-level data; provide overall numbers in this Reporting Summary. Please state if this information has not been collected. Report sex- and gender-based analyses where performed, justify reasons for lack of sex- and gender-based analysis.*
Reporting on race, ethnicity, or other socially relevant groupings	*Please specify the socially constructed or socially relevant categorization variable(s) used in your manuscript and explain why they were used. Please note that such variables should not be used as proxies for other socially constructed/relevant variables (for example, race or ethnicity should not be used as a proxy for socioeconomic status). Provide clear definitions of the relevant terms used, how they were provided (by the participants/respondents, the researchers, or third parties), and the method(s) used to classify people into the different categories (e.g. self-report, census or administrative data, social media data, etc.) Please provide details about how you controlled for confounding variables in your analyses.*
Population characteristics	*Describe the covariate-relevant population characteristics of the human research participants (e.g. age, genotypic information, past and current diagnosis and treatment categories). If you filled out the behavioural & social sciences study design questions and have nothing to add here, write "See above."*
Recruitment	*Describe how participants were recruited. Outline any potential self-selection bias or other biases that may be present and how these are likely to impact results.*
Ethics oversight	*Identify the organization(s) that approved the study protocol.*

Note that full information on the approval of the study protocol must also be provided in the manuscript.

Field-specific reporting

Please select the one below that is the best fit for your research. If you are not sure, read the appropriate sections before making your selection.

[x] Life sciences [ ] Behavioural & social sciences [ ] Ecological, evolutionary & environmental sciences

For a reference copy of the document with all sections, see nature.com/documents/nr-reporting-summary-flat.pdf

Life sciences study design

All studies must disclose on these points even when the disclosure is negative.

Sample size	The sample size was determined based on previous experience with the respective types of experiments; detailed numbers of experiments are indicated in figures and legends.
Data exclusions	No data were excluded from this study. All relevant data are shown.
Replication	All experiments were performed in replicas, the respective numbers are explicitely indicated in text and legends. EM pictograms are representative images.
Randomization	All recordings were performed on randomly selected cells in extended transfection assays.
Blinding	N/A in the reported experiments.

Reporting for specific materials, systems and methods

We require information from authors about some types of materials, experimental systems and methods used in many studies. Here, indicate whether each material, system or method listed is relevant to your study. If you are not sure if a list item applies to your research, read the appropriate section before selecting a response.

Materials & experimental systems

n/a	Involved in the study
☐	☒ Antibodies
☐	☒ Eukaryotic cell lines
☒	☐ Palaeontology and archaeology
☐	☒ Animals and other organisms
☒	☐ Clinical data
☒	☐ Dual use research of concern
☒	☐ Plants

Methods

n/a	Involved in the study
☒	☐ ChIP-seq
☒	☐ Flow cytometry
☒	☐ MRI-based neuroimaging

Antibodies

Antibodies used

- anti-PMCA1-4 ('PMCApan', Invitrogen, Cat.# MA3-914)
- anti-Cav2.2 (Synaptic Systems, Cat. 152303)
- anti-Cav2.1 (described in Althof et al., Front Cell Neurosci 9, 315 (2015))
- anti-NCX2 (Alamone,, Cat. ANX-012)
- anti-IgG (Jackson ImmunoResearch Laboratories)
- anti-IgG (BioCell Research Laboratories)

Validation

Antibodies were verfied in heterologous expressions and in control experiments using target-gene knockout tissue.

Eukaryotic cell lines

Policy information about cell lines and Sex and Gender in Research

Cell line source(s)

- tsa201 nptn/basi double knockout (described in Schmidt et al., Neuron, 96: 827-838 (2017), derived from tsa201, Sigma, Cat#96121229)
- CHO-K1 (Leibniz Institute DSMZ-German Collection of Microorganisms and Cell Culture, Cat#ACC110)
- CHO-K1 at2b2-4 -/-
- CHO cells stably expressing Cav2.2 channels

Authentication

Cell lines were not authenticated.

Mycoplasma contamination

There was no contamination.

Commonly misidentified lines
(See ICLAC register)

Commonly misidentified lines were not used in this study.

Animals and other research organisms

Policy information about studies involving animals; ARRIVE guidelines recommended for reporting animal research, and Sex and Gender in Research

Laboratory animals

Adult C57BL/6 mice (age: 8 weeks), mixed gender (for biochemistry/proteomics), male animals (for EM)

Wild animals

Provide details on animals observed in or captured in the field; report species and age where possible. Describe how animals were caught and transported and what happened to captive animals after the study (if killed, explain why and describe method; if released, say where and when) OR state that the study did not involve wild animals.

Reporting on sex

Indicate if findings apply to only one sex; describe whether sex was considered in study design, methods used for assigning sex. Provide data disaggregated for sex where this information has been collected in the source data as appropriate; provide overall numbers in this Reporting Summary. Please state if this information has not been collected. Report sex-based analyses where performed, justify reasons for lack of sex-based analysis.

Field-collected samples

For laboratory work with field-collected samples, describe all relevant parameters such as housing, maintenance, temperature, photoperiod and end-of-experiment protocol OR state that the study did not involve samples collected from the field.

Ethics oversight

Ethics committee: Regierungspräsidium Freiburg (Germany)

Note that full information on the approval of the study protocol must also be provided in the manuscript.

Plants

Seed stocks	*Report on the source of all seed stocks or other plant material used. If applicable, state the seed stock centre and catalogue number. If plant specimens were collected from the field, describe the collection location, date and sampling procedures.*
Novel plant genotypes	*Describe the methods by which all novel plant genotypes were produced. This includes those generated by transgenic approaches, gene editing, chemical/radiation-based mutagenesis and hybridization. For transgenic lines, describe the transformation method, the number of independent lines analyzed and the generation upon which experiments were performed. For gene-edited lines, describe the editor used, the endogenous sequence targeted for editing, the targeting guide RNA sequence (if applicable) and how the editor was applied.*
Authentication	*Describe any authentication procedures for each seed stock used or novel genotype generated. Describe any experiments used to assess the effect of a mutation and, where applicable, how potential secondary effects (e.g. second site T-DNA insertions, mosiacism, off-target gene editing) were examined.*
